# Temporal transcriptome profiling reveals expression partitioning of homeologous genes contributing to heat and drought acclimation in wheat (*Triticum aestivum* L.)

**DOI:** 10.1186/s12870-015-0511-8

**Published:** 2015-06-20

**Authors:** Zhenshan Liu, Mingming Xin, Jinxia Qin, Huiru Peng, Zhongfu Ni, Yingyin Yao, Qixin Sun

**Affiliations:** State Key Laboratory for Agrobiotechnology, Key Laboratory of Crop Heterosis Utilization (MOE), Beijing Key Laboratory of Crop Genetic Improvement, China Agricultural University, NO.2 Yuanmingyuan Xi Road, Beijing, Haidian District 100193 China

**Keywords:** Wheat, Heat, Drought, Transcriptome, Homeologous genes

## Abstract

**Background:**

Hexaploid wheat (*Triticum aestivum*) is a globally important crop. Heat, drought and their combination dramatically reduce wheat yield and quality, but the molecular mechanisms underlying wheat tolerance to extreme environments, especially stress combination, are largely unknown. As an allohexaploid, wheat consists of three closely related subgenomes (A, B, and D), and was reported to show improved tolerance to stress conditions compared to tetraploid. But so far very little is known about how wheat coordinates the expression of homeologous genes to cope with various environmental constraints on the whole-genome level.

**Results:**

To explore the transcriptional response of wheat to the individual and combined stress, we performed high-throughput transcriptome sequencing of seedlings under normal condition and subjected to drought stress (DS), heat stress (HS) and their combination (HD) for 1 h and 6 h, and presented global gene expression reprograms in response to these three stresses. Gene Ontology (GO) enrichment analysis of DS, HS and HD responsive genes revealed an overlap and complexity of functional pathways between each other. Moreover, 4,375 wheat transcription factors were identified on a whole-genome scale based on the released scaffold information by IWGSC, and 1,328 were responsive to stress treatments. Then, the regulatory network analysis of *HSFs* and *DREBs* implicated they were both involved in the regulation of DS, HS and HD response and indicated a cross-talk between heat and drought stress. Finally, approximately 68.4 % of homeologous genes were found to exhibit expression partitioning in response to DS, HS or HD, which was further confirmed by using quantitative RT-PCR and Nullisomic-Tetrasomic lines.

**Conclusions:**

A large proportion of wheat homeologs exhibited expression partitioning under normal and abiotic stresses, which possibly contributes to the wide adaptability and distribution of hexaploid wheat in response to various environmental constraints.

**Electronic supplementary material:**

The online version of this article (doi:10.1186/s12870-015-0511-8) contains supplementary material, which is available to authorized users.

## Background

Hexaploid wheat (*Triticum aestivum* L. AABBDD), as one of the main food crops, nurtures more than one third of the world population by providing nearly 55 % of the carbohydrates [1, 2]. Environmental constraints, such as extreme high temperature (or heat stress), drought as well as their combination, cause dramatic wheat yield reduction and quality loss which significantly intensify the growing demand of food supply. It is predicted that variation of 2 °C above optimal temperature could lead to wheat yield reductions of up to 50 % *via* perturbations in physiological, biological and biochemical processes [3]. Whereas drought was reported to adversely affect more than 50 % of wheat cultivation area in the world and cause considerable yield loss by inhibiting photosynthesis [4, 5]. Furthermore, drought often occurs simultaneously with high temperature under field condition, and these combined stresses are responsible for a greater detrimental effect on growth and productivity compared to stress applied individually [6–9]. With global warming, extreme high temperature as well as in combination of drought occur more frequently and will be expected to affect crop production more severely [10, 11].

To counter adverse effects of different environmental stresses, plant have evolved special mechanisms and undergone a serial of physiological changes, but the "cross-talk of stresses" and "cross-tolerance to stresses" have not been extensively explored. Some recent studies indicated that both heat and drought stresses reduce plant photosynthetic capacity through chloroplast membrane, thylakoid lamellae damage and metabolic limitation, and combined heat and drought stress decreased photosynthesis efficiency with a greater magnitude than under heat or drought alone and it has been proposed that heat and drought are likely to adversely affect plant growth in a synergistic way rather than a simply additive way of separate stress [7, 12, 13]. However, there are also distinct or even antagonistic responses caused by individual or the combined stresses, e.g. heat stress often leads to stomatal opening to cool leaves by enhancing transpiration while drought usually results in opposite effects and subsequently reduces transpiration capacity, but when subjected to a combination of drought and heat stress, stomata would remain closed and keep a high leaf temperature [12, 14–17]. In addition, some inconsistent physiological results between stress effects have been referred, one study suggests that drought can enhance the PSII tolerance of plants to high temperature, but others reported that drought would exacerbate the sensitivity of heat stress on plant photosynthesis [18, 19]. Thus, our understanding of the interactions between heat and drought stresses, that is, the "cross-talk of stress", is still somewhat ambiguous.

Wheat transcriptome profiling in response to individual stress, such as heat or drought has been investigated [20–23]. However, how the gene expression is regulated to control responses to multiple stresses and finally affect wheat production is not fully understood. In plants, the molecular mechanism underlying tolerance to heat and drought stress combination are best implied from studies of *Arabidopsis*, Tobacco (*Nicotiana tabacum*), *sorghum bicolor* and durum wheat (*Triticum turgidum* subsp. *durum*) [17, 24–26]. It is documented that there is not much similarity of gene responses to heat and drought stress in *Arabidopsis*, and nearly half of differentially expressed genes are specific to combined stress comparing to independent heat or drought stress, including some genes encoding HSPs (heat shock proteins), proteases, starch degrading enzymes, and lipid biosynthesis enzymes [24]. Furthermore, the combination of heat and drought could suppress a proportion of genes which are activated when subjected to individual drought or heat stress in tobacco, such as *dehydrin*, *catalase*, *glycolate oxidase* responding to drought and *thioredoxin peroxidase*, *ascorbate peroxidase* responding to heat [17]. Microarrays analysis of sorghum transcriptome exhibited that the expression of approximately 7 % gene probes were changed only following the combined stress treatment [25]. Rampino et al., (2012) reported that 7, 8 and 15 novel durum wheat genes identified by cDNA-AFLP analysis were up-regulated by heat, drought and their combined stress, respectively. Additionally, transcriptome analysis of wheat caryopses subjected to water shortage alone or combined with heat using 15 k oligonucleotide microarrays revealed that only 0.5 % of the investigated genes were affected by drought alone and a parallel heat treatment increased the ratio to 5–7 % [27]. Transgenic wheat (*Triticum aestivum* L.) lines with overexpression of *betaine aldehyde dehydrogenase* (*BADH*) gene exhibited enhanced tolerance through protecting the thylakoid membrane and promoting antioxidant activity, indirectly increasing photosynthesis and stabilizing water status when exposed to the combination of heat and drought [12, 28]. Together, a subset of genes might only contribute to both drought and heat stress in plants, but till now, limited information is known about this "cross-tolerance to stress" especially in wheat.

Polyploidization has taken place throughout 70 % of angiosperms during their evolutionary history and is thought to have driven more broad adaptability of plants to unpleasant environments [29]. For example, tetraploid *Arabidopsis* exhibited enhanced tolerance to salt stress compared to diploids by elevating leaf K^+^ and reducing leaf Na^+^ accumulation [30]. And a recent study revealed polyploidy *Arabidopsis* decreases transpiration rate and alters the ROS homeostasis, thus improves drought and salt tolerance [31]. However, by what molecular means polyploids accommodating environmental constraints contributes a challenging question. To date, emerging evidences have proposed that subfunctionalization or neofunctionalization of homeologous genes could help account for tolerance to diverse stresses in polyploidy plants. Liu and Adams (2007) reported the function partitioning of the *alcohol dehydrogenase A* gene *AdhA* in allopolyploid cotton (*Gossypium hirsutum*) under abiotic stresses, that is, one copy is only responsive to water-submersion treatment while the other is specifically expressed under cold condition, which might enable polyploidy plants to better cope with stresses in the natural environments [32]. Given that allohexaploid wheat, containing three subgenomes, is widely distributed all over the world, it is likely to possess partitioned expression patterns among homeologous genes responding to biotic or abiotic stresses, but unfortunately, limited information is available to answer this question. In this study, we tried to extensively identify genes responsive to heat stress (HS), drought stress (DS) and their combination (HD) and examine the partitioned expression patterns of homeologous genes under different abiotic stresses in wheat.

## Results

### Transcriptome sequencing, data processing, and reads mapping

To understand transcriptional reprogramming of wheat in response to drought and heat stress, we performed deep RNA sequencing of 1-week old wheat seedling leaves subjected to DS, HS and HD for 1 h and 6 h using the Illumina sequencing platform. After removing reads with low-quality, a total of approximately 900 million 100 bp paired-end reads were generated, with an average of 66 million filtered reads for each library including DS-1 h, DS-6 h, HS-1 h, HS-6 h, HD-1 h, HD-6 h and control, respectively (see Methods, Additional file 1).

Due to unavailability of complete wheat genome information that possibly resulted from high levels of repetitive sequences or insufficient reads coverage, up to 30 % reads could not be mapped to current wheat genome released by International Wheat Genome Sequencing Consortium (IWGSC) [33]. This issue potentially leads to a missing report of many stress associated genes. Thus, to minimize this influence and map an informative, stress-related wheat transcriptome, we combined gene sequences collected from both public databases (including IWGSC, NCBI Unigene Database, and TriFLDB as well) and our *de novo* assembly, and in total, 109,786 non-redundant wheat unigenes were identified, consisting of 81,308 genes from IWGSC, 14,298 *de novo* transcripts from our assembly and 14,180 mRNA sequences from other public databases (Additional file 2).

Next, the high-quality reads of 14 samples were mapped to the reference sequences by Bowtie2, and only uniquely mapped reads were retained for the following expression analysis by edgeR [34, 35] (Additional file 1). Finally, we identified 29,395 differentially expressed genes in wheat seedling leaves in at least one stress condition compared to control (fold change ≥2 and false discovery rate (FDR) adjusted *p* <0.01) (Additional file 3).

### Global comparisons of DS, HS and HD related transcriptomes reveal their complexity and overlapping

**Fig. 1:**
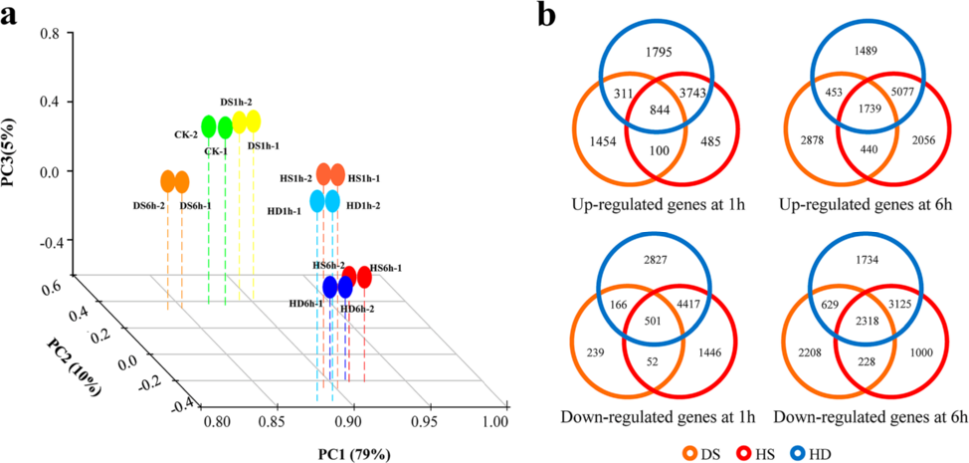
Comparative analysis of transcriptome profiles of wheat seedling leaves under DS, HS and HD. (**a**) Principal component analysis (PCA) of mRNA populations from control, DS-1 h, DS-6 h, HS-1 h, HS-6 h, HD-1 h and HD-6 h, each sample contained two replicates. Principal components (PCs) 1, 2 and 3 account for 79 %, 10 % and 5 % of the variance, respectively. PCA plot shows two distinct groups of mRNA populations. Group I: CK (green), DS-1 h (yellow) and DS-6 h (brown); Group II: HS-1 h (light red), HS-6 h (dark red), HD-1 h (light blue) and HD-6 h (dark blue). (**b**) Venn diagrams showing overlap of up- or down-regulated genes in response to the three assayed abiotic stresses at 1 h and 6 h: drought (yellow), heat (red) and combined stress (blue)

To provide a framework to understand how wheat genes are regulated to respond stresses, we first compared mRNA populations from all transcriptomes globally using principal component analysis (PCA, Fig. 1a). Transcriptomes of HS-1 h and HD-1 h as well as HS-6 h and HD-6 h were likely to share a great similarity in overall gene expression, respectively, which formed two groups that were far deviated from the control. While transcriptomes of DS exhibited distinct relationship from that of HS and HD, suggesting a major shift in gene expression occurred in DS responsive transcriptome compared with HS and HD.

Comparison of differentially expressed genes responding to DS, HS and HD further supports our observation in the PCA analysis (Fig. 1b). Among the up- or down-regulated genes, the overlap of HS and HD was significantly higher than that of DS and HD, with the proportion of 52-63 % compared to 8-29 %. In addition, approximately 46.2 % and 46.7 % of differentially regulated genes were uniquely responsive to DS-1 h and DS-6 h, respectively, rather than HS or HD (Fig. 1b). Specifically, we identified 8,732 (including 2,709 for DS-1 h, 5,172 for HS-1 h and 6,693 for HD-1 h) and 14,132 (including 5,510 for DS-6 h, 9,312 for HS-6 h and 8,758 for HD-6 h) up-regulated genes plus 9,648 (including 958 for DS-1 h, 6,416 for HS-1 h and 7,911 for HD-1 h) and 11,242 (including 5,383 for DS-6 h, 6,671 for HS-6 h, 7,806 for HD-6 h) down-regulated genes after stress treatment at 1 h and 6 h, respectively, and observed a higher proportion of stress responsive genes at 6 h compared to that at 1 h regardless of DS, HS or HD (Additional file 4). In addition, 6566, 10,441, 10,771 and 5348, 9,704, 11,006 genes were significantly up- and down-regulated, respectively, when exposed to DS, HS and HD at either time point (Additional file 4). Interestingly, although HD shared a great similarity with DS or HS in terms of stress-related genes (approximately 64 ~ 83 %), there were still 1,738 (16 % of HD up-regulated genes) and 2,482 genes (23 % of HD down-regulated genes) exhibiting specific responses to the stress combination (Additional file 4). Taken together, the results suggest that DS responsive transcriptomes differ fundamentally from that of HS and HD, and they show complex relationships dependent on a temporal cue. Furthermore, the combination of heat and drought stress might activate HD-specific functional pathways to counteract with multiple effects.

### DS, HS and HD responsive genes encode distinct functional groups

**Fig. 2:**
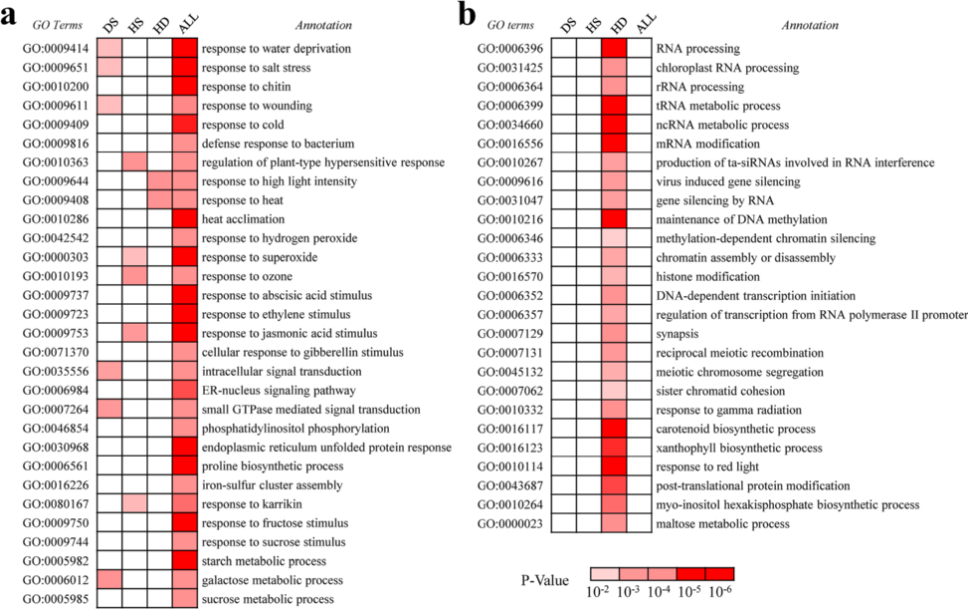
Heat map showing the *P* value significance of enriched GO categories for DS, HS and HD responsive genes. (**a**) Functional enrichment analysis indicates that GO terms related to responses to abiotic stress and hormones were over-presented in DS, HS and HD commonly up-regulated genes. (**b**) GO terms associated with RNA processing and epigenetic regulation of gene expression were enriched in HD specifically up-regulated genes. The color scale in white (low, *p-value* ≥ 10^−2^), pink (medium, 10^−4^ < *p-value* < 10^−2^), and red (high, *p-value* ≤ 10^−4^) represents the relative *P* value significance which is determined by Fisher’s exact test

Although an overlap, a set of stress responsive genes exhibited altered expression patterns specific to DS, HS and HD, indicating distinguished functional categories could be involved in response to different stresses. Therefore, we performed Gene Ontology (GO) enrichment analysis to examine the functional distribution of the stress related genes identified in our study (Fig. 2; Additional file 5). A serial of GO categories exhibited significantly higher enrichments in the overlapped, up-regulated gene sets (*p* < 0.01) under DS, HS and HD treatments compared to the background. These groups mainly included GO terms of stress response, hormone stimulus response and nutrient metabolic processes (Fig. 2a). Moreover, except for the abiotic stress related GO terms, biotic stress related GO term e.g. "defense response to bacterium (GO:0009816)" also exhibited significant enrichment among these commonly up-regulated genes (Fig. 2a). All the above evidences collectively suggest that wheat shared a "cross-tolerance" in the molecular functions responsive to heat, drought and their combination, and possibly biotic stress.

Of the stress responsive GO terms, two distinct functional categories of HD specifically up-regulated genes exhibited significantly higher enrichments compared to the individual stress (*p* < 0.01), namely RNA processing and epigenetic regulation of gene expression (Fig. 2b). The first group included "chloroplast RNA processing (GO:0031425)", "rRNA processing (GO:0006364)", "tRNA metabolic process (GO:0006399)" and "ncRNA metabolic process (GO:0034660)", whereas the second group contained "methylation dependent chromatin silencing (GO:0006346)", "maintenance of DNA methylation (GO:0010216)", "chromatin assembly or disassembly (GO:0006333)", "histone modification (GO:0016570)" for transcriptional regulation, "production of ta-siRNAs involved in RNA interference (GO:0010267)", "virus induced gene silencing (GO:0009616)", "gene silencing by RNA (GO:0031047)" for post-transcriptional regulation (Fig. 2b). Overall, these functional categories indicated that epigenetic modifications might play a crucial role in the HD responsive process, although the exact functions of these genes remain to be elucidated. However, previous studies have reported that H3K23ac and H3K27ac modifications on the H3 N-tail are correlated with gene activation of drought stress-responsive genes and RNA-dependent DNA methylation pathway is required for the basal heat tolerance of *Arabidopsis* on a transcriptional level [36, 37], so we propose that the roles of epigenetic modification in heat and drought stress responses need to be further explored. It is also worthy noticing that these conclusions confirmed the observation that the combination of heat and drought exceedingly complicates the corresponding molecular pathways compared to separate stress, rather than a simply additive effect.

To determine the potential functions of down-regulated genes by DS, HS or HD, we also applied GO enrichment analysis on them and observed distinct functional categories enriched in down-regulated genes compared with that of up-regulated genes (Additional file 5). The commonly down-regulated genes by DS, HS and HD were mainly enriched in two GO groups including photosynthesis and nutrient biosynthesis pathway, suggesting a cross-talk among these abiotic stresses which adversely affect wheat growth through similar pathway. For HD specifically down-regulated genes, several other GO categories uniquely exhibited higher enrichments compared to the background, e.g. "vesicle mediated transport" and "regulation of cell cycle process" (Additional file 5). Therefore, our RNA-Seq data suggested that different abiotic stresses could influence wheat growth in a cross-talk manner, while wheat might trigger similar functional pathways responding to different stresses in a cross-tolerance manner. Besides, the combination of heat and drought stress act in a synergistic way and may control specific cellular or biochemical processes compared to individual stress based on our analysis.

### Identification of temporally up- and down-regulated transcription factors (TFs) in response to DS, HS and HD

TFs have been demonstrated to play master roles in response to various abiotic stresses *via* modulating target gene expression [38, 39]. To understand the nature of regulatory processes during DS, HS and HD treatment, we first predicted wheat transcription factors on a whole-genome scale based on our identified 109,786 non-redundant wheat unigenes by using a domain searching method [40]. In total, 4,375 wheat TF genes distributed among 51 families were identified (Additional file 6), compared to 1,940 TFs released in Plant TFDB (Additional file 7) [40], providing a more comprehensive wheat TF database for our following analysis.

**Fig. 3:**
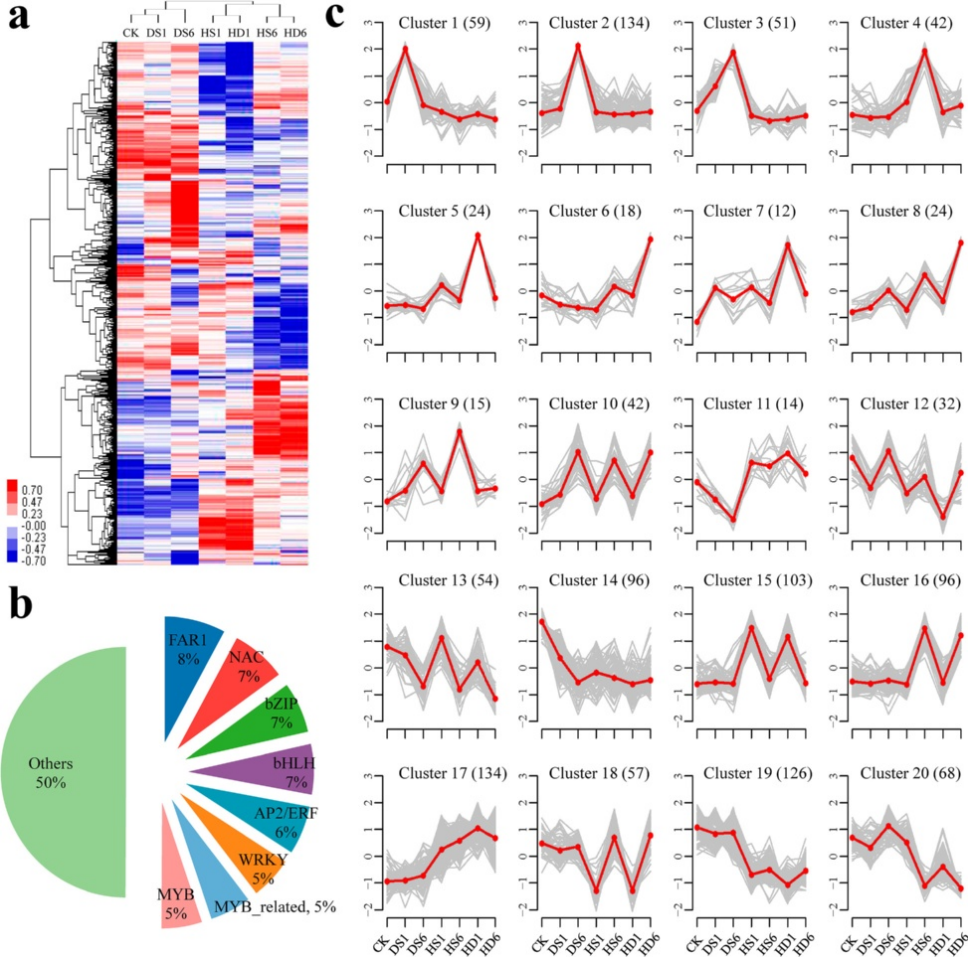
Clustering analysis of DS, HS and HD responsive TFs. (**a**) Hierarchical clustering of TFs with altered expression levels in response to DS, HS and HD at 1 h and 6 h. The color scale of blue (low), white (medium) and red (high) represents the normalized expression levels of differentially expressed TFs. (**b**) Pie chart showing top 7 TF families which contain approximately 50 % of differentially expressed TF genes. (**c**) Clustering of the differentially expressed TFs based on their expression patterns in response to DS, HS and HD at 1 h and 6 h. 20 clusters comprising of 1,187 TFs are exhibited here, the numbers in parentheses indicate TF amount in corresponding clusters. X axis represents treatment conditions and y axis represents centralized and normalized expression value. The red lines represent the mean expression trend of TFs (gray lines) belonging to each cluster

To profile stress responsive TFome under DS, HS and HD, we focused on TF genes exhibiting diverse expression patterns with temporal changes, including continuous up-regulation, continuous down-regulation, an early peak of expression and a late peak of expression patterns, and found 1,328 TFs distributed in 50 families were differentially regulated in response to at least one stress (fold change ≥ 2 and FDR adjusted *p* < 0.01) (Fig. 3a; Additional file 6 and 8). Among which, seven TF families accounted for approximately half of stress responsive TF genes, including FAR1 (8 %), NAC (7 %), bZIP (7 %), bHLH (7 %), AP2/ERF (6 %), WRKY (5 %), Myb-related (5 %) and Myb (5 %) (Fig. 3b).

Next, we further classified these 1,328 TFs into 20 clusters according to their expression patterns by performing Mfuzz program in R software [41] (Fig. 3c; Additional file 9 and 10). Cluster 1, 2 and 3, consist of 244 TFs mainly up-regulated by DS (Fig. 3c), including five genes encoding *DREB1A* (two, two and one in cluster 1, 2 and 3, respectively) which have been proved to be key factors in plant drought resistance pathway [42, 43]. We also observed a TF gene encoding a bZIP protein, homologous to *ABF3* in *Arabidopsis*, also presented in this group, and constitutive expression of *ABF3* enhanced expression of ABA-responsive genes e.g. *RD29B*, *RA18*, *ABI1* and *ABI2*, leading to enhanced survival under severe water deficit in *Arabidopsis*, rice, lettuce (*Lactuca sativa*) and creeping bentgrass (*Agrostis tolonifera* L.) [44–48]. Interestingly, six homologs of *Arabidopsis HSFC1* showed DS specifically induced expression patterns either at 6 h or at both time points. Meanwhile, among HS predominantly induced genes (Cluster 4, Fig. 3c), four genes encoding Auxin Response Factors (ARFs, homologues to *ARF6* and *ARF8* in *Arabidopsis*) were identified, indicating auxin could be involved in wheat responses to heat stress. Consistently, exogenous application of auxin can completely reverse male sterility and recover normal seed setting rate of *Arabidopsis* and barley under increasing temperatures [49, 50], although Min et al. (2014) reported that high concentration of auxin might be a disadvantage for cotton anther development during heat stress [51].

Cluster 5, 6, 7 and 8, representing a total of 77 TFs, were preferentially up-regulated by the combination of heat and drought (Fig. 3c). Of these genes, two TFs encoded heat shock factors similar to HSFA3, which was shown to be directly up-regulated by DREB2A and DREB2C and required for the basal and acquired thermotolerance in *Arabidopsis* [52–54]. In contrast, TFs in cluster 9 exhibited different expression trends that they were up-regulated by both DS-6 h and HS-6 h but not HD (Fig. 3c), including homologs of *INDUCER OF CBP EXPRESSION 1* (*ICE1*) and *RAP2.6 L. Arabidopsis ICE1*, encoding a MYC-type basic helix-loop-helix (bHLH) transcription factor, has been reported to confer chilling and freezing tolerance by directly regulating *CBF3/DREB1A* expression and activating downstream cold responsive genes [55–57]. Overexpression of *RAP2.6 L* in *Arabidopsis* can enhance tolerance to salt, drought and also waterlogging stress possibly *via* mediating several stress hormones signaling pathways like abscisic acid, jasmonic acid, salicylic acid, and ethylene [58, 59].

Among the down-regulated TF genes by DS, HS and HD (cluster 12–14, 18–20), a large proportion were noticed to be involved in the regulation of plant growth and development. For example, a gene annotated as a member of *PLETHORA* family (*PLT3*) in cluster 19 is essential for phyllotaxis development by controlling local auxin biosynthesis [60, 61]. Interestingly, TFs in cluster 12 draw our particular attention because these stress responsive genes exhibited a dynamic expression pattern at different time points and the extent of down-regulation was much more pronounced in HD-1 h compared to DS and HS. Except for plant growth regulators such as *BPC6*, *KANADI2* (*KAN2*) and *ARR12* which were well documented to play important roles in a range of developmental processes in *Arabidopsis*, this cluster contained a transcriptional repressor named *NAC Transcription factor-like 9* (*NTL9*). Silencing of *NTL9* increased resistance to the bacterial pathogen *Pseudomonas syringae* DC3000, and overexpression of *NTL9* in transgenic lines reduced disease resistance in *Arabidopsis* [62]. Together, this analysis described a dynamic stress responsive TF transcriptome landscape in wheat seedling leaf and provided an opportunity to identify co-expressed TF gene sets that represent regulatory nodes participating in the regulation of wheat responses to DS, HS and HD.

### *HSFs* and *DREBs* regulated complicated and partially overlapped gene networks in response to DS, HS and HD

**Fig. 4:**
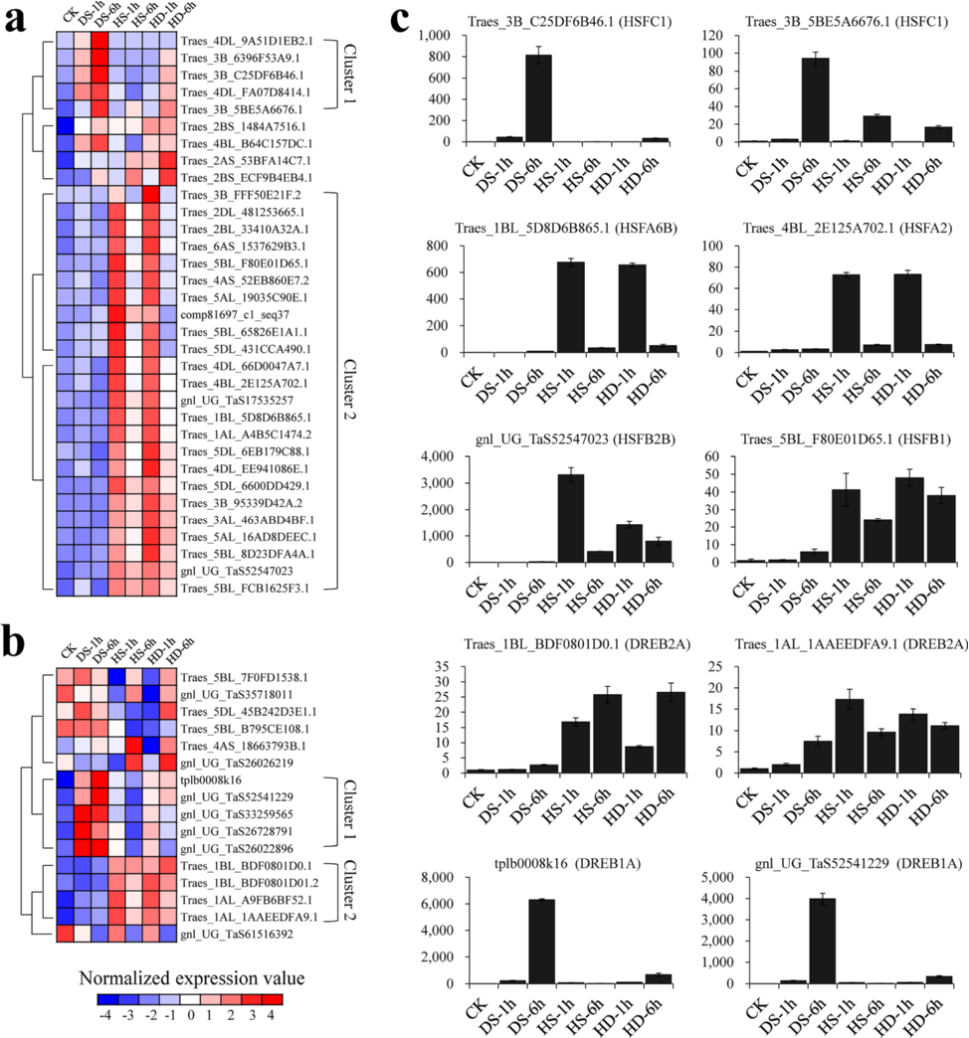
Hierarchical clustering and quantitative analysis of *HSFs* and *DREBs’* expression in response to DS, HS and HD. (**a**) Heat map showing the expression patterns of stress responsive *HSFs.* Two specific groups of *HSFs* exhibiting DS/HD or HS/HD up-regulated expression patterns including five and 24 *HSF* genes respectively, were identified. (**b**) Heat map showing the expression patterns of stress responsive *DREBs.* Two specific groups of *DREBs* exhibiting DS/HD or HS/HD up-regulated expression patterns including five and four *DREB* genes respectively, were identified. (**c**) Experimental validation of 10 randomly selected *HSFs* and *DREBs* by using quantitative RT-PCR. The expression patterns of two cluster1-*HSFs*, four cluster2-*HSFs*, two cluster1-*DREBs* and two cluster2-*DREBs* were validated after DS, HS and HD treatments at 1 h and 6 h, which exhibited similar expression patterns compared to the results revealed by RNA-Seq data

Plant responses to environmental limiting factors are regulated by extensive transcriptional regulatory networks that trigger specific gene expressions [63–65]. Understanding how the transcriptional reprograms are orchestrated by TFs at a molecular level is an essential step towards deciphering the mechanisms underlying DS, HS or HD tolerance of wheat. Thus, we developed a framework to predict the interacting modules of TFs and their co-expressed, potential target genes. Two groups of *HSF*s and *DREB*s were selected as central genes to analyze the regulatory circuitry (Fig. 4a and b), because they were well known to participate in the regulation of heat or drought responsive genes and associates with definite *cis*-acting elements [43, 66, 67]. Moreover, they exhibited interesting expression patterns that *DREBs*-group1 and *HSFs*-group1 showed induced expression trends when subjected to DS and HD, whereas *DREBs*-group2 and *HSFs*-group2 showed up-regulated expression patterns when encountering HS and HD. To confirm their expression patterns, 10 out of 38 candidates were validated by quantitative RT-PCR (Fig. 4c; Additional file 11).

**Fig. 5:**
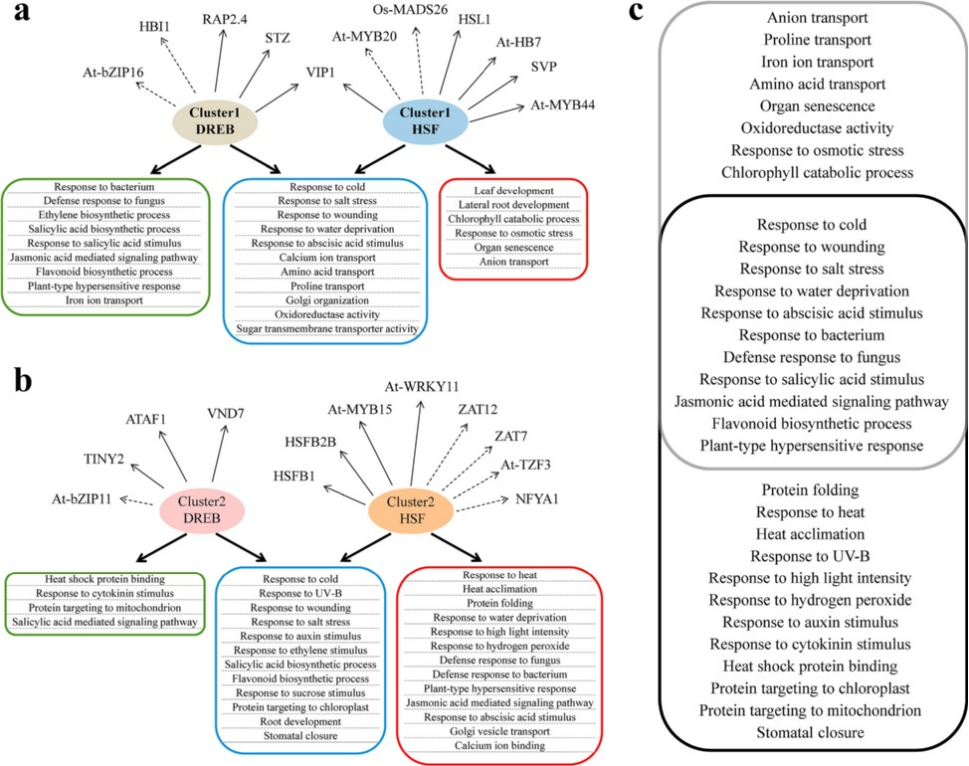
Predicted transcriptional modules regulating wheat responses to DS, HS and HD. (**a**) GO terms (rounded rectangle) that are significantly overrepresented (*p* < 0.01, Fisher's exact test) within the DS/HD induced *DREBs* and *HSFs* co-expressed gene sets. Green rounded rectangle represents specific functional categories enriched in Cluster1-*DREBs* potentially regulated genes, red for Cluster1-*HSFs* and blue for both. A proportion of co-expressed transcription factors are also represented, arrows with solid lines indicate those TFs have been reported to be involved in drought stress responses, whereas dash lines represent TFs conferring tolerance to other abiotic stresses. (**b**) GO categories that are significantly overrepresented (*p* < 0.01, Fisher's exact test) within the HS/HD induced *DREBs* and *HSFs* co-expressed gene sets. Green rounded rectangle represents specific functional categories enriched in Cluster2-*DREBs* potentially regulated genes, red for Cluster2-*HSFs* and blue for both. A proportion of co-expressed transcription factors are also represented, arrows with solid lines indicate those TFs have been reported to be involved in heat stress responses, whereas dash lines represent TFs conferring tolerance to other abiotic stresses. (**c**) Comparison of GO categories enriched in two groups of predicted *DREBs* or *HSFs* target genes. Almost half of GO categories were shared by both groups*.* Gray rounded rectangle contains GO terms belonging to Group1 and black rounded rectangle contains GO terms belonging to Group2

In total, 305 *DREBs*-group1 and 678 *HSFs*-group1 co-expressed genes with respective binding motifs in their promoter regions were identified, among which, 123 were potentially commonly regulated by both types of TFs. Comparison of GO enrichments of these two groups of activated genes revealed that 11 functional categories were shared between each other, including response to abiotic stress (water deprivation, wounding, cold and salt stress), transport (proline, calcium and amino acid) and oxidoreductase activity *etc.* (Fig. 5a)*.* In addition, we observed nine and six GO categories exhibiting significantly higher functional enrichments specific to *DREBs*-group1 and *HSFs*-group1 up-regulated genes, respectively. The former category mainly included response to biotic stresses and hormone, while the latter associated with plant development (Fig. 5a). Previous studies found that several TFs, up-regulated by *DREBs*-group1 or *HSFs*-group1, have been verified to play central roles in drought resistance, e.g. *RAP2.4*, a member of *DREB* subfamily A-6, confers enhanced tolerance to drought stress in a ABA-independent way by inducing *RD29A*, *COR47*, and *COR15A* [68]. Whereas *STZ* and *HB-7*, acting as growth repressors, contributed to drought resistance in a ABA-dependent pathway in *Arabidopsis*, although constitutive expression of *STZ* and *HB-7* under CaMV35S promoter caused growth retardation (Fig. 5a) [69–71].

Correspondingly, 258 *DREBs*-group2 and 825 *HSFs*-group2 up-regulated genes were characterized when subjected to HS and HD including 105 overlapped. GO enrichment analysis of these genes revealed complex and interesting functional terms that, like group1, "abiotic stress response" categories were commonly enriched in these genes. Surprisingly, besides "response to heat" and "heat acclimation", "response to water deprivation" category was also enriched in *HSFs*-group2 up-regulated genes while "heat shock protein binding" enrichment was observed among *DREBs*-group2 regulated genes, indicating there might be direct or indirect interactions between the two TF families in response to HS and HD (Fig. 5b), which is similar to the reports that *DREB2A* and *DREB2C* are able to interact with the promoter of *HSFA3* as activators, subsequently promote the expression of heat shock proteins and enhanced tolerance to HS in *Arabidopsis* [52–54]. It should be noted that heat shock factors are probably regulated by themselves based on our co-expression analysis (Fig. 5b). This is also supported by binding element analysis in previous studies that HsfA1a and HsfA1b interact with each other in vivo in *Arabidopsis* examined by bimolecular fluorescence complementation and immunoprecipitation assay [72–74]. Furthermore, we compared the enriched GO terms in up-regulated genes by the two groups of TFs, and observed approximately half of functional categories were present in both classes indicating wheat responses to HS and DS were closely connected on the molecular level (Fig. 5c).

### A large proportion of wheat homeologous genes exhibited differential responses to DS, HS and HD

As an allohexaploid, bread wheat contains three subgenomes, namely, A, B and D, and shows improved tolerance to salt, low pH, aluminum, and frost compared to tetraploid [29]. However, the mechanisms underlying this broader adaptability are still ambiguous. With the support of our high-throughput RNA sequencing and informative homeolog SNPs identified by using the available information of 21 chromosomes released by IWGSC, we are able to distinguish the origins and quantify the expression of homeologous genes from three subgenomes. To minimize artifacts from incomplete genome assembly, we only focused on 4,565 homeologous gene loci that had exactly one representative member from each subgenome (referred to as homeologous triplets; 4565 × 3 = 13,695 genes) in the following analysis (Additional file 12) and quantified their expression according to A-unique, B-unique and D-unique reads (Methods, Additional file 13), which enable us to examine the homeologous gene expression patterns in response to DS, HS and HD. We first performed a Fisher’s exact test to determine whether the ratio of each homeologous loci derived reads significantly deviated from the expect ratio of 1A:1B:1D in normal condition (control). At a significance level of *p* = 0.01, 63.9 % (2,916/4,565 triplets) homeologous genes exhibited unequal contribution to total transcription level in both replicates. Next, we narrowed the list of candidate genes using more stringent criteria to precisely reflect the biased expression status of the homeologous genes, namely, the maximum expression level should be at least 1.5 fold of the minimum expression level (Exp_max_/Exp_min_ ≥ 1.5) in terms of SNP-associated reads that mapped to a homeologous locus. Finally, the ratio-based cutoff shortened the list to 2,270 triplets (49.7 %) with biased expression between three homeologous loci in untreated samples.

**Fig. 6:**
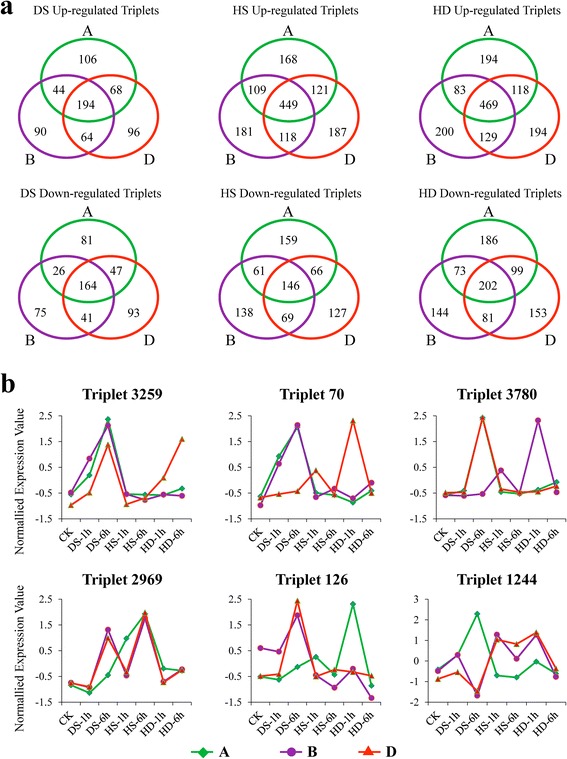
Expression partitioning analysis of homeologous genes in response to DS, HS and HD. (**a**) Venn diagram showing the partitioned expression patterns of homeologous genes in response to DS, HS and HD. Green circle: subgenome A, purple circle: subgenome B, red circle: subgenome D. (**b**) The expression partitioning of homeologs exhibited temporal and stress-specific patterns. Green line: A-homeolog, purple line: B-homeolog, red line: D-homeolog. Triplet 3259, homolog of AT5G63650, encoding *SNF1-RELATED PROTEIN KINASE 2* (*SnRK2*); Triplet 126, homolog of AT2G18950, encoding *homogentisate phytyltransferase 1* (*HPT1*); Triplet 3780, homolog of AT5G64530 encoding xylem NAC domain 1 protein (*XND1*); Triplet 2969, homolog of AT2G42750, encoding a DNAJ heat shock N-terminal domain-containing protein; Triplet 70, homolog of AT5G50170, encoding a GRAM domain containing protein; Triplet 1244, homolog of AT4G24380, encoding an alpha/beta-Hydrolases superfamily protein

Subsequently, we identified 2,804 differentially expressed triplets (with at least one homeolog gene differentially expressed) out of 4,565 by comparing their expression levels between stress and normal conditions (fold change ≥ 2, FDR adjusted *p* < 0.01). Specifically, 412 (318), 847 (432) and 864 (560) A-homeologs were up-regulated (down-regulated) under DS, HS and HD, 392 (306), 857 (414) and 881 (500) for B-homeologs, and 422 (345), 875 (408) and 910 (535) for D-homeologs, respectively (Fig. 6a). Furthermore, to examine partitioned expression of homeologs in response to stress treatments, we first classified these homeologous triplets into two groups based on their expression level in untreated sample as described above, that is, triplets with equal contribution (ECTs) or unequal contribution (UCTs) between homeologous loci in the control (including 1,109 and 1,695, respectively) (Additional file 14). Then, we compared the changing trends between wheat homeologs responding to stresses, namely, calculating the ratio of fold change between A-, B- and D-homeologs subjected to DS, HS and HD (e.g. A_HS/CK_/B_HS/CK_). Of the 1,109 ECTs, 617 triplets exhibited differentially expression trends under at least one stresses with the criteria of two fold change, accounting for approximately 55.6 %, and correspondingly, the proportion is about 76.7 % (1,300/1,695) for UCTs (Additional file 14). Therefore, on average, 68.4 % of homeologs exhibited differential expression patterns after stress in wheat. Moreover, we clustered these triplets into 12 distinct categories based on partitioned expressions between A-, B- and D-homeologs (Additional file 15). Interestingly, the expression partitioning of homeologs exhibited temporal or stress-specific patterns (Fig. 6b). For example, the D-homeolog of Triplet 3259 (*SNF1-RELATED PROTEIN KINASE 2, SnRK2*) was specifically up-regulated under HD-6 h compared to A- and B-homeolog, although all of three were abundantly expressed at HS-6 h. Similarly, A-homeolog of Triplet 126 (homogentisate phytyltransferase, *HPT1*) exhibited peak expression at HD-1 h compared to the other two. Interestingly, it has been reported that *SnRK2* and *HPT1* were involved in drought stress response through ABA signaling pathway and tocopherol biosynthesis, respectively [75, 76]. In addition, Triplet 3780, encoding a NAC transcription factor XND1, was proved to negatively regulate lignocellulose synthesis and programmed cell death in xylem [77]. Homeologs of Triplet 3780 showed partitioned expression trends and only B copy exhibited high expression level when subjected to HD-1 h, while the other two copies were abundantly expressed at 6 h after drought stress. Likewise, Triplet 2969 (chloroplast J protein, known as co-chaperone of Hsp70), Triplet 70 (GRAM domain containing protein) and Triplet 1244 (alpha/beta-Hydrolases) also exhibited differential expression patterns between homeologs in response to stresses (Fig. 6b).

**Fig. 7:**
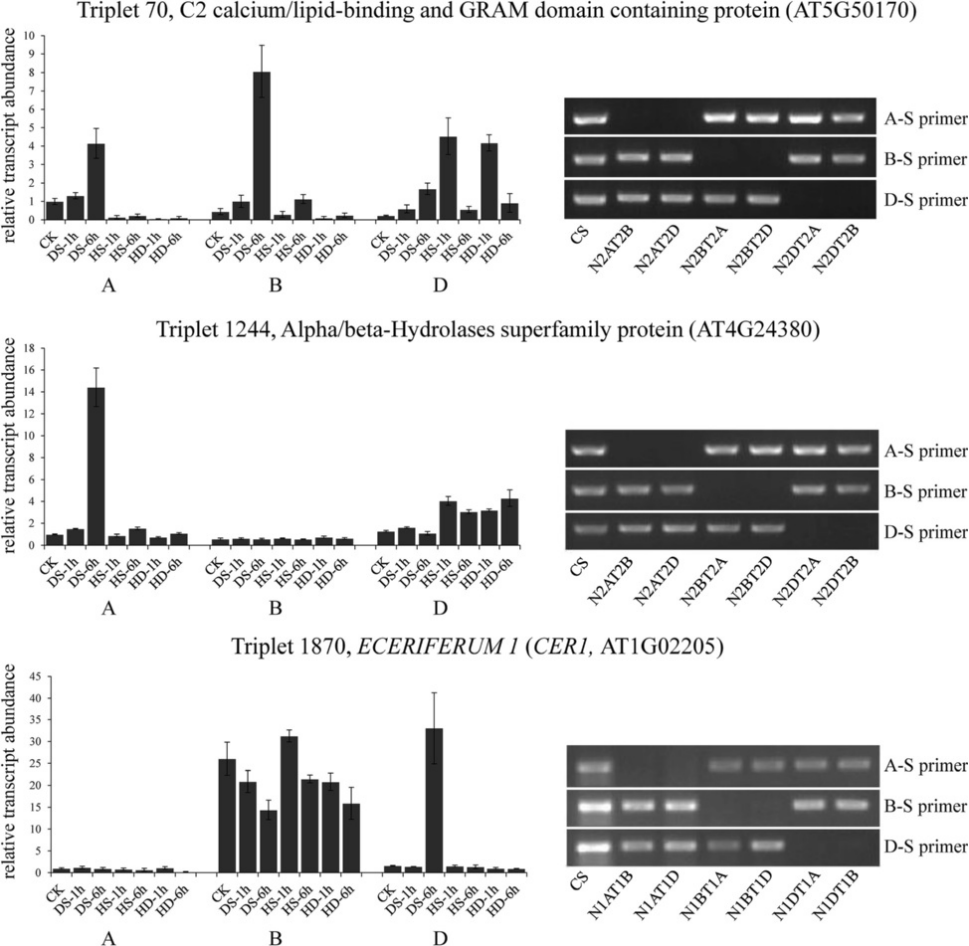
Expression and chromosome location analysis of wheat homeologous genes by using primer-specific quantitative RT-PCR and Nullisomic-tetrasomic lines. The partitioned expression of homeologs in Triplet 70, 1244 and 1870 validated by qRT-PCR exhibited similar changing trends with RNA-Seq data, and Nullisomic-tetrasomic lines validation using homeologous gene specific primers further confirmed their localization on the corresponding subgenomes. A-S: A-homeolog specific, B-S: B-homeolog specific, D-S: D-homeolog specific

To further confirm the partitioned expression patterns of UCTs and their responses to different stress treatments as well as subgenome locations, nine triplets (Triplet 722, 272, 1681, 2282, 765, 3766, 70, 1244 and 1870) were examined by using Nullisomic-Tetrasomic lines and primer-specific qRT-PCR. Nullisomic-Tetrasomic line detection indicated our primers were homeolog specific and qRT-PCR results showed their expression partitioning was consistent with our observation obtained from RNA-seq data (Additional file 16, Fig. 7). Both the qRT-PCR and RNA-Seq analysis documented differential expression patterns of A-, B- and D-homeolog under normal condition, and reveled their distinct responses to heat, drought or their combination stress (Additional file 16). Specifically, B-homeolog of Triplet 1244 was specifically silenced in all samples and A-homeolog was particularly induced by DS-6 h, whereas D-homeolog was responsive to both HS and DS albeit their relative low abundance (Fig. 7). Similarly, the expression of A-homeolog of Triplet 1870 was silenced, while the abundance of D-homeolog was specifically induced when encountering DS-6 h, however, its B-homeolog did not exhibit any significant differences after stress treatments, even if it was expressed at a high level in all samples (Fig. 7). Interestingly, Triplet 1870 was annotated as *Arabidopsis ECERIFERUM1* (*CER1*) which was proposed to be involved in a major step of wax production and directly impacts drought resistance of *Arabidopsis* and rice [78–80]. The expression patterns of Triplet 70 were more complex: A- and B-homeolog exhibited most abundant expression at 6 h after DS while its D-homeolog was up-regulated mainly by HS and HD at 1 h (Fig. 7).

## Discussion

### Heat and drought stress are likely to interact with each other in a synergistic manner

Plants, being sessile, have evolved to develop specific and complex mechanisms in response to different abiotic stresses at transcriptome, cellular and physiological levels. Several lines of evidences have indicated that, rather than being simply additive, the way how plants respond to combined stresses occurred in the field is largely distinct compared with individual stress applied in the laboratory, and the complicated interactions of heat and drought (cross-talk of stresses) and orchestrated plant responses to these stresses (cross-tolerance to stress) are still ambiguous.

In addition, how heat and drought combination together prevent wheat growth and reproduction is not fully explored. Rizhsky *et al*. (2002) reported that DS and HS may have conflicting responses, for example, plants prefer to open stomata to cool their leaves by enhancing transpiration under heat condition, but in contrast, stomata will remain closed if DS and HS occur simultaneously, leading to a high temperature of leaves, which supports "stress matrix" hypothesis that heat and drought have a potentially negative interaction [81]. However, other studies indicated that DS and HS influence each other in a synergistic way that they will greatly exacerbate the adverse effects on plant growth and photosynthesis compared with individual stress alone [10, 19, 82]. Recently, Pradhan *et al.* (2012) proposed that the interaction between drought and heat stress was hypo-additive by analyzing yield loss of synthetic hexaploid wheat and spring wheat cultivars under DS, HS and HD at anthesis stage, that is, the yield loss caused by combined stress is higher than individual stress but lower than their sum, assuming that both of the stresses negatively regulate partial physiology, growth and yield traits in common [7]. Thus, the molecular mechanisms underlying cross-talk of stresses on plants and cross-tolerance of plants to stresses are still unclear, but our study provides a new perspective towards understanding these processes and interactions from transcriptional level.

Our transcriptome analysis of wheat seedling leaves subjected to abiotic stresses (DS, HS and HD at 1 h and 6 h, respectively) exhibited that approximately 64.3 % to 82.9 % genes were commonly up- or down-regulated between combined stress and individual stress (Fig. 1b), which supports the hypothesis proposed by Pradhan *et al.* (2012) that these three stresses may influence a proportion of genes in common and inhibit plant growth and production together. Furthermore, GO analysis confirmed this observation that a set of functional pathways were commonly regulated by DS, HS and HD, for instance, response to abiotic stress (water deprivation, heat, wounding and salt), response to hormone (ABA, JA, ethylene and GA) and carbohydrate metabolism categories were all enriched in commonly up-regulated genes, whereas GO terms related to photosynthesis were enriched in commonly down-regulated genes (Fig. 2a; Additional file 5). In addition, we also identified a group of differentially expressed genes (approximately 17 % to 35.7 %) specifically responding to HD (Fig. 1b), which is consistent with reports in *Arabidopsis* that stress combination requires a unique acclimation response that are not altered by drought or heat stress alone [24]. Besides, the transcriptome profiling documented that the acclimation responses of wheat to DS and HS are distinguished and only a small overlap of responsive genes were observed between each other, and correspondingly, DS and HS particularly triggered activation or suppression of thousands of genes respectively (Fig. 1b; Additional file 4), which may explain why the adverse effects caused by combined stress is not simply additive effects of individual stress. Interestingly, we observed that a large proportion of TFs (Cluster5-8, 12 and 17) were up- or down-regulated to a more pronounced level in HD compared with DS and HS (Fig. 3c), suggesting combined stress might have a synergistic interaction in adversely affecting wheat growth and development. However, we did not observe a clear antagonistic interactions between heat and drought based on our GO analysis, which were further confirmed by analyzing *DREBs* and *HSFs* regulated stress-responsive genes between DS and HS (Fig. 5), although nearly half of the functional categories were distinct (Fig. 5c). Taken together, our transcriptome sequencing analysis suggests that the concurrence of heat and drought stress will not only alter expression profiles of partial individual stress responsive genes but also trigger activation or depression of a proportion of HD specific genes, leading to a complicated gene regulatory network in wheat acclimation response to drought and heat combination.

### A subset of *DREBs* and *HSFs* up-regulated genes may not necessarily contribute to stress tolerance

In plant genome, there are approximately 7 % of the coding sequences encoding TFs and they play a central role in regulating gene responses to abiotic and biotic stresses at molecular level [38, 64, 83]. In total, we predicted 4,375 potential TFs on wheat genome, accounting for approximately 4.6 % of total genes, and the number is two times higher compared to the 1,940 TFs registered in plantTFDB, although the proportion is less than the expected 7 % [40].

*DREBs* and *HSFs* have been demonstrated to be master regulators of gene networks in plant acclimation response to drought and heat by regulating responsive gene expressions *via* binding to the *cis*-acting elements DRE (dehydration-responsive element) and HSE (heat shock sequence elements) [67, 84]. Consistent with the expectation, our results suggest the functions of *DREB* and *HSF* family members have undergone diversification during wheat evolution and both of them can confer stress tolerance in wheat by activating comprehensive GO categories, ranging from abiotic stresses response, hormone response to morphogenesis (Fig. 5). But unexpectedly, some validated negative stress-regulators were also up-regulated by *DREBs* or *HSFs* under DS or HS condition, which make it more complicated to understand the molecular mechanisms underlying wheat tolerance to abiotic stress. For example, *VIRE2-INTERACTING PROTEIN 1* (*VIP1*), a bZIP transcription factor, rapidly enhances the expression of *CYP707A1/3* by directly binding to their promoter regions and then inactivates ABA by catalyzing its catabolic pathway (Fig. 5a), finally, represses ABA responsive genes and attenuates plant tolerance to abiotic stress [85]. This information indicates that even a positive regulator may not necessarily regulate genes all contributing to stress tolerance, a certain set of stress sensitive genes may also be activated and attenuate stress tolerance. This finding suggests that researchers should be very careful when improving plant tolerance to multiple stresses by manipulating a single TF gene due to its "side effects". Thus our results indicate TFs regulated cross-tolerance to abiotic stress in wheat is considerably complex, but it is helpful for us to understand the cellular and molecular mechanisms underlying wheat tolerance to multiple simultaneous stresses and develop broad-spectrum stress-tolerant crops, although difficult.

### Expression partitioning of homeologous genes may facilitate abiotic acclimation of wheat

Polyploidization is a major driving force in plant evolution, which contributes greatly to a large number of duplicated genes (termed as homeologs) [86–88]. As a prominent model system to study polyploidy, bread wheat arose from hybridization between the allotetraploid cultivated *Triticum turgidum* (2n = 4x = 28, AABB) and the diploid wild goat grass *Aegilops tauschii* (2n = 2x = 14, DD), followed by spontaneous chromosome doubling approximately 8,000 years ago [89–91]. Thus, bread wheat comprise three diploid homeologous chromosome sets (A, B and D), and theoretically, every gene should be represented by three homeologs on wheat genome. However, allopolyploid often undergo extensive genomic rearrangements by the "genome shock", causing physical loss of a large fraction of homeologs and subsequently leading to functional differentiation [92, 93]. Therefore, the expression of homeologs in allopolyploid wheat is prone to partition ranging from slight alteration to complete absence of expression, indicative of subfunctionalization [88, 94, 95]. Consistently, 55 % of wheat genes were reported to be only expressed from one or two homeologous loci in root and shoot due to genome sequence loss or transcriptional silencing [96]. In addition, greater gene silencing was observed in chromosome 7A and 7B compared to chromosome 7D, and only 1,291 out of 2,386 (approximately 54 %) genes exhibited expression from all three homeologous loci, which further confirmed gene expression partitioning among wheat homeologous genes [97]. A detailed study of wheat gene *LEAFY HULL STERILE1* (*WLHS1*) exhibited that only *WLHS1*-D functions in hexaploid wheat due to a large fragment insertion in *WLHS1*-A causing its dysfunction and high cytosine methylation on *WLHS1*-B leading to its predominant silencing [98]. In addition, Hu et al. (2013) reported that permanent silencing of *TaEXPA1-*B gene is closely associated with altered DNA methylation in bread wheat [99]. Moreover, a fraction of expressed homeologs in allopolyploids are likely to respond differently when subjected to stresses. For example, Dong and Adams (2011) investigated the expression patterns of homeologs in response to heat, cold, drought, high salt and water submersion stresses in allotetraploid cotton (*Gossypium hirsutum*) by using SSCP analysis and documented that 23 out of 30 examined genes (approximately 77 %) exhibited variation in the contribution of homeologous genes to abiotic stresses possibly due to epigenetic modification or regulatory region variation [100]. Carvalho et al. (2014) also found the homeologs of the *Coffea canephora* involved in mannitol pathway presented unequal contribution in response to drought, salt and heat stresses [101]. Besides, the expression of *AdhA* gene homeologs in allotetraploid cotton diverged significantly under multiple stresses and showed reciprocal silencing of homeologs in response to water submersion and cold stress, respectively, indicating subfunctionalization in response to abiotic stress conditions [32]. It is also reported that homeologs of wheat *MBD* (*methyl CpG-binding domain protein gene*) gene contribute differentially in response to cold and salt stress with a high expression level of *TaMBD2*-B compared to the other two [102]. Therefore, it is reasonable to speculate that a proportion of homeologs would contribute differentially when subjected to environmental limiting factors.

Although partitioned expression of homeologs, up to now, there is little information about analysis of their expression divergence on a genome-wide level in wheat, especially under stress conditions. Our analysis of wheat leaf transcriptome reveals that approximately 68.4 % of homeologs have differential expression patterns under DS, HS or HD condition. But compared with allotetraploid cotton, we observed that wheat has a relatively lower proportion of homeologous genes with unequal contribution under stress (~68.4 % vs. 77 %), one possible reason is that Dong and Adams (2011) examined only a subset of homeologs which might not be generally applicable when applied to the whole *G. hirsutum* transcriptome as the author mentioned. However, all the evidences above collectively suggest that abiotic stress related subfunctionalization might have occurred during wheat evolution based on the hypothesis that different expression patterns probably mean different functions, but more efforts are needed to verify this phenomenon. Yet, our study provides a new perspective to understand the broad adaptability and worldwide distribution of hexaploid common wheat [29] which might be partially explained by the observation of 'complementary response' of homeologs to different stresses at different time-points. For example, A-homeolog of triplet 2969 exhibited high expression level at both 1 h and 6 h after HS while B-, D-homeolog were up-regulated by both DS and HS but only at 6 h (Fig. 6b), which might enable wheat to counteract various environmental constraints in a lasting period. Overall, this analysis indicated that gene expression partitioning in response to abiotic stress is a common phenomenon in wheat, which can be considered as an orchestrated co-operation between homeologous genes drove by evolution force and may contribute greatly to stress acclimation, and help to explain why there are about 70 % of angiosperm plants have experienced one or more episodes of polyploidy during their evolutionary histories [103–105].

## Conclusions

Our results revealed that the combination of heat and drought stress act in a synergistic manner rather than a simply additive way, and a group of genes involved in specific cellular or biochemical processes were only responsive to combined stress but not individual heat or drought. In addition, a large proportion (68.4 %) of wheat homeologous genes exhibited partitioned gene expression in a temporal and stress-specific manner when subjected to DS, HS and HD. Taken together, this study deepens our understanding of the complicated interactions of heat and drought (cross-talk of stresses) and orchestrated wheat responses to the combined stress (cross-tolerance to stress), which frequently occurred under field condition and provides a new perspective to understand the broad adaptability and worldwide distribution of hexaploid common wheat. To our knowledge, this is the first study to explorer the differential contributions of homeologous genes to abiotic stress response in hexaploid wheat on a genome-wide scale. Therefore, our study will contribute to the current body of knowledge on subfunctionalization of homeologous genes in wheat.

## Methods

### Plant materials and stress treatments

TAM107 is a leading wheat variety during late 1980's and early 1990's in western Kansas, which was released by Texas A&M University in 1984 [106] and it developed a reputation for both heat and drought tolerant (Wheat Genetics Resource Center, Kansas) [107]. Seeds of the wheat cultivar ‘TAM 107’ were surface-sterilized in 1 % sodium hypochlorite for 20 min, rinsed in distilled water for six times, and soaked in dark overnight at room temperature. The germinated seeds were transferred into Petri dishes with filter paper and cultured in water (25 seedlings per dish, one biological replicate), and five independent biological replicates were employed, with two for sequencing and the other three for experimental verification. Prior to stress treatments, the seedlings were grown in a growth chamber with 22 °C/18 °C (day/night), 16 h/8 h (light/dark), and 50 % humidity, then the seedlings were subjected to heat stress (40 °C), drought stress (20 % (m/V) PEG-6000) and combined heat and drought stress (40 °C and 20 % PEG-6000) for 1 h and 6 h, respectively. Drought stress was applied by replacing water with 20 % PEG solution and roots were totally covered by PEG solution [108, 109]. Heat stress was applied by moving the plants to another growth chamber with 40 °C temperature. All experiments were performed in parallel and seedlings in normal growth condition (22 °C, well watered) were taken as control. Leaves were collected separately at 1 h and 6 h after stress treatment and frozen immediately in the liquid nitrogen, and stored at −80 °C for further use.

### RNA isolation, library preparation and transcriptome sequencing

The total RNA from leaf tissues was extracted using TRIzol reagent (Invitrogen), according to the manufacturer’s instructions. RNA concentration was measured using a NanoDrop 2000 spectrophotometer (ND-2000, Thermo Fisher Scientific, Inc., USA). RNA integrity was assessed on an Agilent 2100 Bioanalyzer (Agilent Technologies, Inc., CA, USA). Paired end (PE) sequencing libraries with average insert size of 200 bp were prepared with TruSeq RNA Sample Preparation Kit v2 (Illumina, San Diego, USA) and sequenced on HiSeq2000 (Illumina, San Diego, USA) according to manufacturer’s standard protocols. Raw data obtained from Illumina sequencing were processed and filtered using Illumina pipeline (http://www.Illumina.com) to generate FastQ files. Finally, approximately 184.3G high quality 100-bp pair-end reads were generated from 14 libraries (Additional file 1).

### *De novo* sequence assembly

To obtain a high quality transcriptome assembly, a strict filtering criteria was employed to filter sequencing reads, that is, any bases with a low Phred quality score (<15) were trimmed from 3’- or 5’-end of reads and reads with averagely high Phred quality score (>20) were retained. After processing, approximately 80 % (152.5 Gb out of 184.3 Gb) of high-quality sequencing data were left for *de novo* assembly, which was carried out by running Trinity with the following parameters ‘--seqType fq --JM 200G --CPU 24 --group_pairs_distance 550 --min_kmer_cov 2’ [110]. To improve efficiency, we performed a Perl script normalize_by_kmer_coverage.pl in Trinity software package (with the parameters ‘--seqType fq --max_cov 30 --PARALLEL_STATS --pairs_together’) before running Trinity. Totally, 630,618 transcripts distributed in 116,653 trinity components (multiple alternatively spliced transcripts from a gene locus) were obtained with average length of 1,454 bp and N50 length of 2,100 bp, and the longest transcript of each trinity component was selected as representative for the construction of wheat unigenes dataset (Additional file 2).

### Alignment of RNA-Seq reads and expression analysis

The high quality paired-end RNA-Seq reads from each library were aligned to wheat reference sequences including unigenes identified from wheat genome sequences released by IWGSC (accessible at http://plants.ensembl.org/Triticum_aestivum), NCBI Wheat UniGene Build #62, TriFLDB [111] and our *de novo* assembly by Bowtie2 with the parameters ‘-5 5–3 5 --no-unal -a -phred33 --end-to-end -X 600 --reorder --score-min L,-0.6,-0.3 -L 15’ [34]. Reads uniquely mapped to the reference sequences (with ≤1 mismatch) were used for differential expression analysis which was performed by using edgeR package (ver. 3.2.3) in R software (ver. 3.0.1) with criteria of fold change ≥2 and false discovery rate (FDR, Benjamini and Hochberg's method) adjusted *p* <0.01 [35].

### Heatmap and principal component analysis (PCA)

Hierarchical clustering analysis of the expression data of genes was performed based on average linkage clustering with Cluster 3.0 [112]. Heatmaps demonstrating the gene expression data were created by the Java TreeView [113]. Principal component analysis was performed using ‘principal’ fuction in R software (ver. 3.0.1). And PCA plots among the biological replicates are generated by ‘scatterplot3d’ package in R software (ver. 3.0.1).

### Prediction of *HSF* and *DREB* target genes

The *HSF* binding *cis*-regulatory element (HSE) ‘GAANNTTC’ and ‘TTCNNGAA’ were obtained from Stress Responsive Transcription Factor Database (STIFDB V2.0) [114], and the *DREB* binding *cis-* regulatory element (DRE) ‘(A/G/T)(A/G)CCGACN(A/T)’ was obtained from *Arabidopsis* Gene Regulatory Information Server (AGRIS) [115]. Due to the incompleteness of wheat genome sequence released by IWGSC, only genes with a start codon according to the genome annotation were used for following analysis and 2 kb upstream sequences of the first exon were used for searching HSE and DRE motifs by a custom Perl script. Then, the expression patterns of genes with HSE or DRE motifs were examined and only those with similar expression trends compare with *HSFs* and *DREBs* were considered as *HSFs* or *DREBs* co-expressed genes and used for network analysis.

### Homeologous genes expression analysis

The flowchart of homeologous gene expression analysis was shown in Additional file 13. Wheat genes of A-, B- and D-subgenome from IWGSC were compared against each other by using BLASTN (e-value cutoff 1e-10) considering only alignments with minimum 75 % sequence coverage and 90 % sequence similarity [116]. After that, all the aligned sequences were clustered and we only retained clusters that had exactly one representative member from each subgenome and located on similar position of homeologous group. Therefore, only homeologous gene loci that had exactly one representative member from each subgenome (referred to as homeologous triplets, 4565 × 3 = 13,695 genes) (Additional file 12 and 17) were selected for further analysis.

The high quality paired-end RNA-Seq reads from each library were mapped to triplets by Bowtie2 [34] with the parameters ‘-5 5–3 5 --no-unal --no-hd -a --phred33 --end-to-end --ignore-quals -L 15 --mp 6,6 --rfg 7,6 --rdg 7,6 --score-min L,-0.6,-0.63 -reorder’ and only reads mapped to all three homeologs were retained for following analysis. Then, these reads were divided into 10 groups depending on the SNPs information between the homeologs (Additional file 13). Next, the reads counts originating from each homeolog were calculated based on the mapped reads of these 10 groups. Reads that map ambiguously to two or three homeologs were divided proportionally based on the counts of A, B and D specific reads (Additional file 13).

We compared the expression of A-, B- and D-homeologs under normal condition pairwise (A vs B, A vs D, B vs D). If one of the three comparison showed significant difference (Fisher’s exact test, *P*-value < 0.01) and the ratio of maximum expression value of the three homeologs to the minimum was greater than or equal to 1.5 (Exp_max_/Exp_min_ ≥ 1.5), the homoeologous gene loci was defined as UCT (triplets with unequal contribution), otherwise, the gene loci was defined as ECT (triplets with equal contribution).

### Quantitative real time PCR (qRT-PCR) validation

DNase I treated total RNAs were reverse transcribed with oligo-dT primers using Reverse Transcriptase M-MLV (TaKaRa, Japan), following the manufacturer's instructions. qRT-PCR was performed in a 10 μl reaction volume using CFX96 Real-Time PCR Detection System (Bio-Rad Laboratories, Inc., USA) with SYBR Green PCR master mix (TaKaRa, Japan), and three biological replicates were conducted for each reaction. Wheat *Actin* (5’-GACCGTATGAGCAAGGAGAT-3’ and 5’-CAATCGCTGGACCTGACTC-3’) was used as an internal reference gene to normalize Ct values of each reaction, which were determined using the CFX96 software with default settings. The primers used in qRT-PCR analysis were listed in Additional file 18.

### Availability of supporting data

The RNA-Seq reads used for this study are deposited at the National Center for Biotechnology Information Short Read Archive (http://www.ncbi.nlm.nih.gov/sra/) under accession number SRP045409.

## Additional files

Additional file 1: Table S1. Summary of RNA-Seq data and reads mapping. Additional file 2: Fig. S1. Flowchart of identification of Wheat Unigene Dataset. 109,786 wheat unigenes were identified from public sequence information released from IWGSC, NCBI, TriFLDB and our de novo assembly. Additional file 3: Table S2. Differentially expression of 29,395 stress responsive genes under DS, HS and HD. Additional file 4: Fig. S2. The number of differentially expressed genes under DS, HS and HD. **(a)** Number of differentially expressed genes in response to 1 h and 6 h of DS, HS and HD. In total, 29,395 wheat genes were differentially expressed under at least one stress condition. **(b)** Venn diagram exhibited an overlap of these stress responsive genes between DS, HS and HD (at either time point). Additional file 5: Fig. S3. GO Categories enriched in stress-specifically and commonly responsive genes. **(a)** GO Categories enriched in DS and HS specifically up-regulated genes. The color scale represents the relative P value significance which is determined by Fisher’s exact test. **(b)** GO Categories enriched in DS, HS and HD specifically and commonly down-regulated genes. Additional file 6: Fig. S4. Prediction of wheat transcription factors. On the whole genome level, 4,375 wheat transcription factors were identified based on our identified 109,786 non-redundant wheat unigenes, among which, 1,328 were differentially expressed when subjected to DS, HS or HD. Additional file 7: Table S3. Comparison of our predicated TFs with that released by PlantTFDB. Additional file 8: Table S4. Statistics of differentially expressed TFs under DS, HS and HD. Additional file 9: Table S5. Distribution of differentially expressed TFs among the 20 clusters. Additional file 10: Table S6. Detail lists of 20 clusters of differentially expressed transcription factors. Additional file 11: Fig. S5. The expression patterns of HSFs and DREBs under stress conditions revealed by RNA-seq data. The expression trends of HSFs and DREBs determined by RNA-seq and qRT-PCR are consistent, indicating the high confidence of RNA-seq data. Additional file 12: Table S7. List of identified 4,565 homeologous triplets. Additional file 13: Fig. S6. Flowchart of homeologous gene expression analysis. **(a)** Identification of triplets based on wheat reference genes released by IWGSC. In total, 4,565 triplets were identified based on our criteria, and 2,804 were differentially expressed when subjected to DS, HS or HD. **(b)** Expression analysis of A-, B- and D-homeologs. Reads mapped to a triplet can be classified into 10 groups based on SNP information and the formulas used to calculate each homeolog’s expression are shown. Additional file 14: Table S8. Details of 2,804 differentially expressed triplets. Additional file 15: Fig. S7. Clustering analysis of stress-related homeologs with differential responses. (a) Triplets showing differential responses between homeologs can be clustered into 12 clusters based on homeologs responsive patterns. The red and blue line of each chart represents the average responsive trend of up- and down-regulated homeologs, respectively. (b) Statistics of the numbers of triplets within the 12 clusters. Additional file 16: Fig. S8. Validation of A-, B- and D-homeolog expression profiles of UCTs revealed by RNA-Seq. (a) Comparison of A-, B- and D-homeolog expression profiles revealed by RNA-Seq and qRT-PCR. X axis represents A-, B- and D-homeolog under different treatment conditions and y axis represents normalized expression value. Red line: qRT-PCR data, Blue line: RNA-seq data. Triplet 722, homolog of AT1G63680 encoding an acid-amino acid ligase; Triplet 272, homolog of AT3G47690 encoding microtubule end binding protein EB1A; Triplet 1681, homolog of AT3G10370 encoding glycerol-3-phosphate dehydrogenase SDP6; Triplet 2282, homolog of AT1G05675 encoding an UDP-Glycosyltransferase superfamily protein; Triplet 765, homolog of AT1G02850 encoding beta glucosidase 11; Triplet 3766, homolog of AT1G53210 encoding a sodium/calcium exchanger family protein. The partitioned expression of homeologs in Triplet 722, 272, 1681, 2282, 765 and 3766 validated by qRT-PCR exhibited similar changing trends with RNA-Seq data. (b) Homeolog-specific primer verification. Nullisomic-tetrasomic lines were used to validate primer specificity and homeologs' localization. AS: A-homeolog specific, BS: B-homeolog specific, DS: D-homeolog specific. Additional file 17:
**Sequences of 4,565 homeologous triplets.**
Additional file 18: Table S9. Primers used in qRT-PCR analysis.

## Abbreviations

DS: Drought stress
HS: Heat stress
HD: Heat and drought combined stress
GO: Gene ontology
IWGSC: International Wheat Genome Sequencing Consortium
RT-PCR: Quantitative real time PCR
PSII: Photosystem II
HSPs: Heat shock proteins
cDNA-AFLP cDNA: Amplified fragment length polymorphism
BADH: Betaine aldehyde dehydrogenase
ROS: Reactive oxygen species
AdhA: Alcohol dehydrogenase A
PCA: Principal component analysis
TF: Transcription factor
ABF3: Abscisic Acid Responsive elements-binding Factor 3
RD29B: Responsive to Dessication 29B
ABI1: ABA insensitive 1
ABI2: ABA insensitive 2
HSF: Heat shock factors
ICE1: Inducer of CBP Expression 1
RAP2.6 L: Releted to AP2 6 L
PLT3: PLETHORA 3
BPC6: Basic Pentacysteine 6
KAN2: KANADI2
ARR12: Arabidopsis response regulator 12
NTL9: NAC Transcription factor-like
RAP2.4: Related to AP2 4
RD29A: Responsive to Dessication 29A
COR47: COLD-REGULATED 47
COR15A: COLD-REGULATED 15A
STZ: Salt Tolerance Zinc finger
HB-7: HOMEOBOX 7
SNP: Single Nucleotide Polymorphism
ECTs: Triplets with equal contribution
UCTs: Triplets with unequal contribution
HPT1: Homogentisate phytyltransferase
SnRK2: SNF1-RELATED PROTEIN KINASE 2
XND1: Xylem NAC domain 1
CER1: ECERIFERUM1
JA: Jasmonic acid
GA: Gibberellic acid
DRE: Dehydration-responsive element
HSE: Heat shock sequence elements
VIP1: VIRE2-interacting protein 1
WLHS1: Wheat LEAFY HULL STERILE1
EXPA1: Expansin 1
SSCP: Single-strand conformation polymorphism
MBD: Methyl CpG-binding domain protein gene
PEG-6000: Polyethylene glycol 6000
