# Supplementary figures and images for: Temporal transcriptome profiling reveals expression partitioning of homeologous genes contributing to heat and drought acclimation in wheat (Triticum aestivum L.)
Source: BMC Plant Biol. 2015 Jun 20;15:152. doi: 10.1186/s12870-015-0511-8 (PMC4474349; doi:10.1186/s12870-015-0511-8)

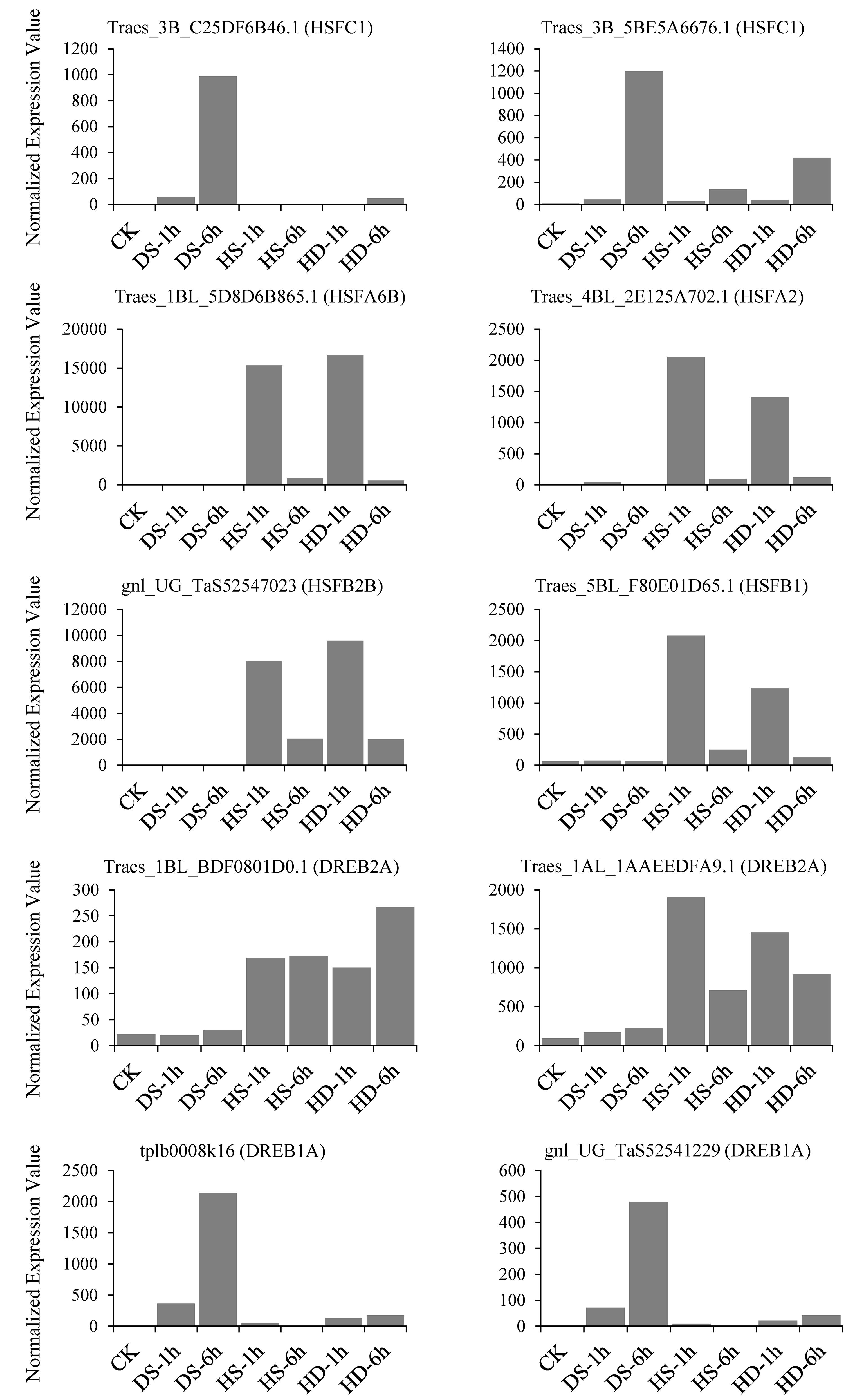

Supplement: Additional file 2: Fig. S1. — Flowchart of identification of Wheat Unigene Dataset. 109,786 wheat unigenes were identified from public sequence information released from IWGSC, NCBI, TriFLDB and our de novo assembly. [file 12870_2015_511_MOESM11_ESM.tiff]

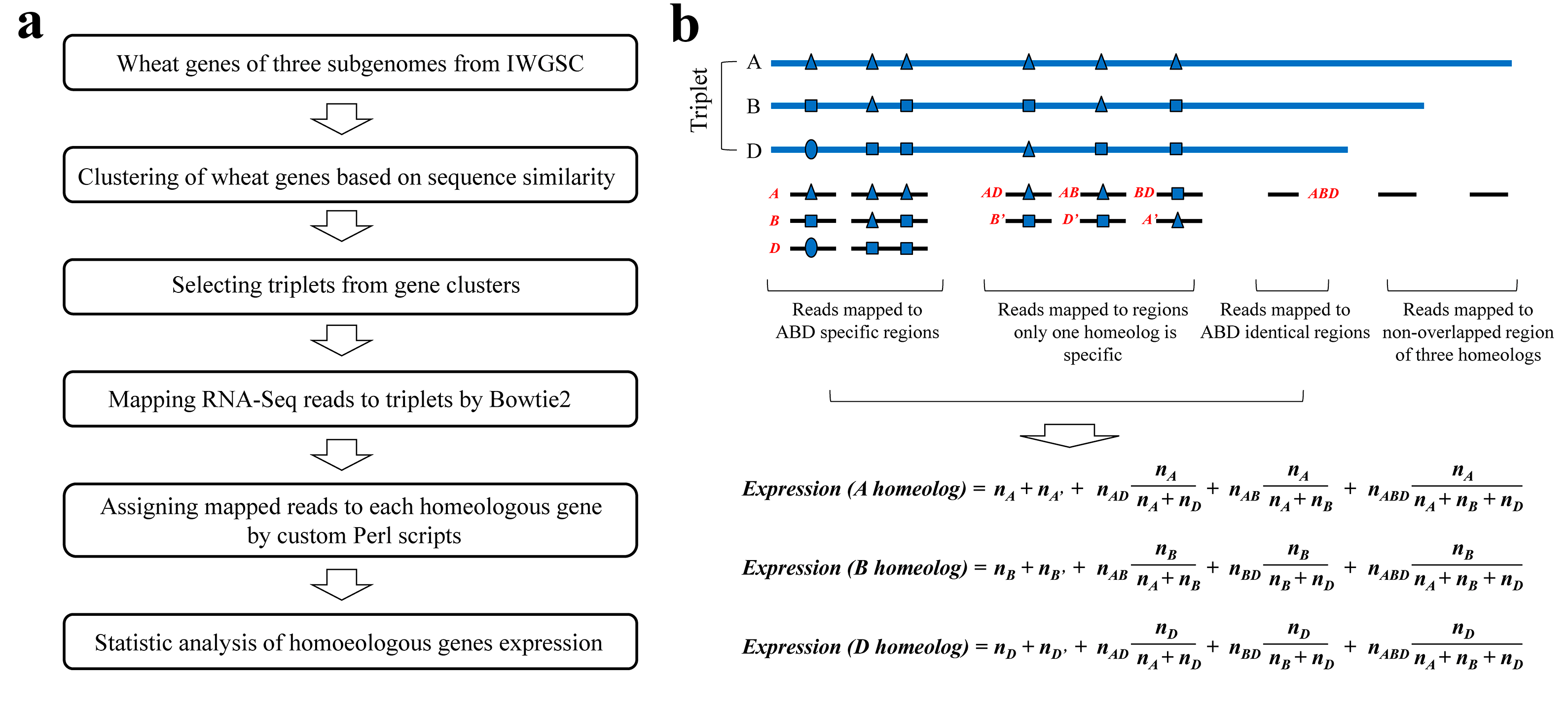

Supplement: Additional file 4: Fig. S2. — The number of differentially expressed genes under DS, HS and HD. (a) Number of differentially expressed genes in response to 1 h and 6 h of DS, HS and HD. In total, 29,395 wheat genes were differentially expressed under at least one stress condition. (b) Venn diagram exhibited an overlap of these stress responsive genes between DS, HS and HD (at either time point). [file 12870_2015_511_MOESM13_ESM.tiff]

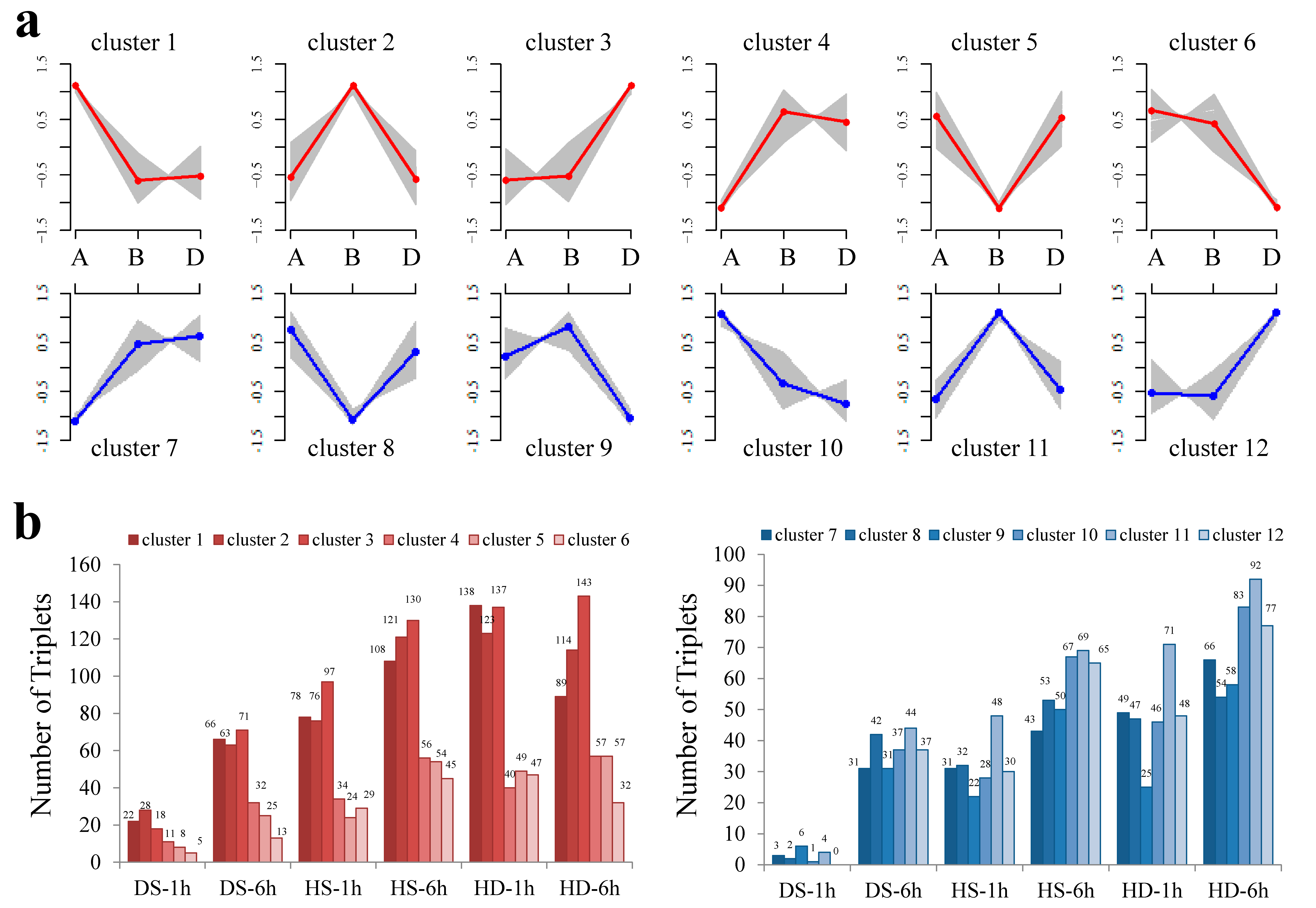

Supplement: Additional file 6: Fig. S4. — Prediction of wheat transcription factors. On the whole genome level, 4,375 wheat transcription factors were identified based on our identified 109,786 non-redundant wheat unigenes, among which, 1,328 were differentially expressed when subjected to DS, HS or HD. [file 12870_2015_511_MOESM15_ESM.tiff]

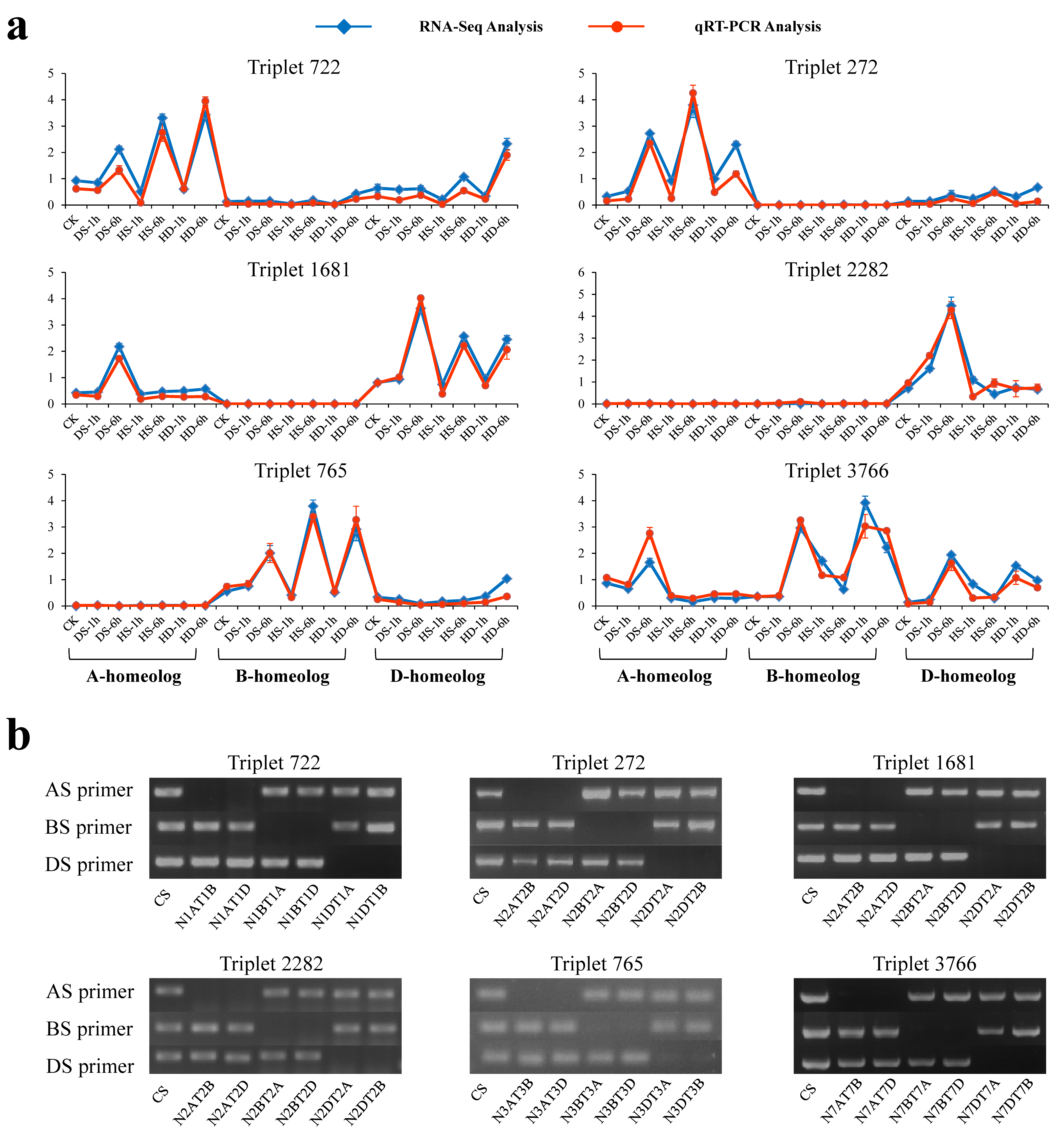

Supplement: Additional file 7: Table S3. — Comparison of our predicated TFs with that released by PlantTFDB. [file 12870_2015_511_MOESM16_ESM.tiff]

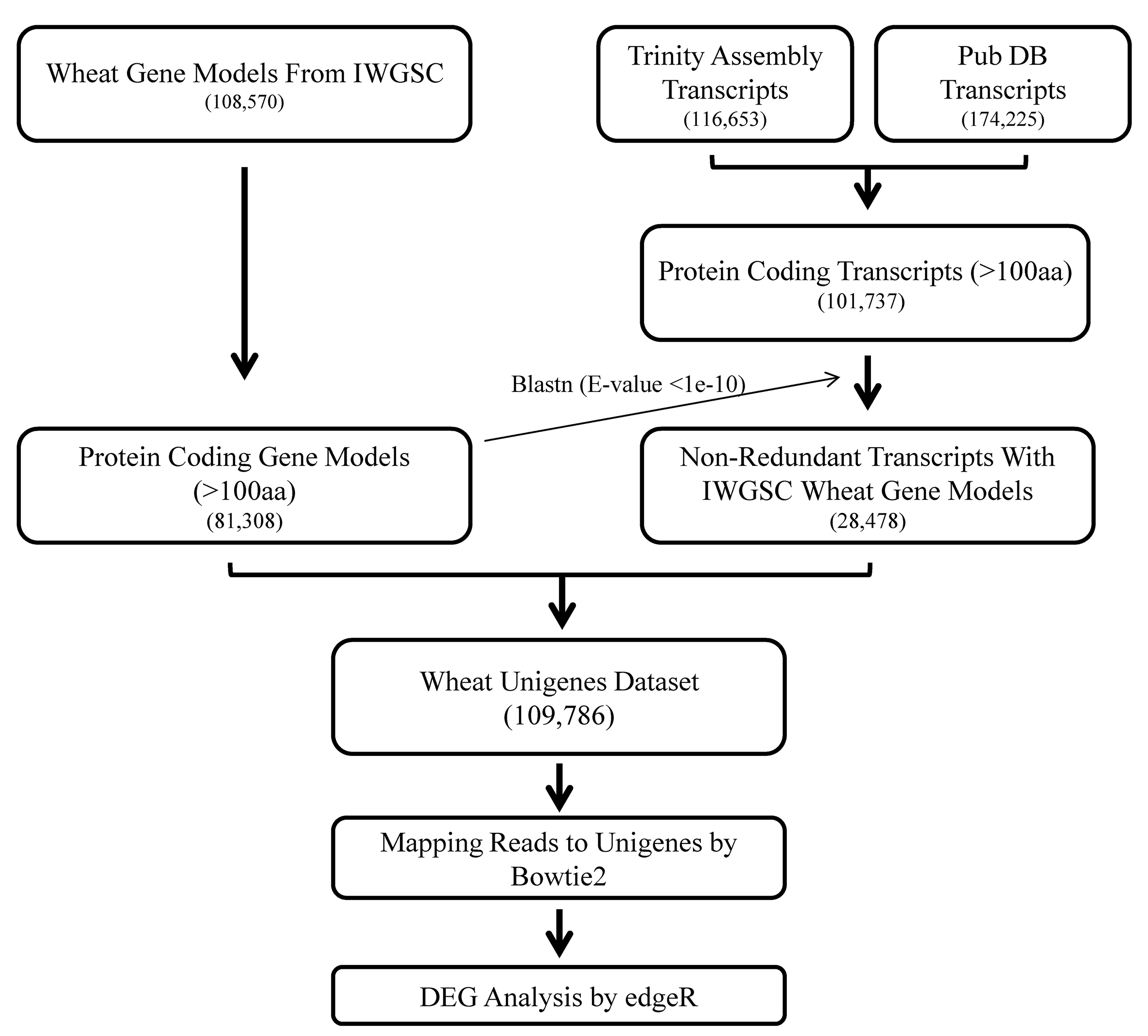

Supplement: Additional file 11: Fig. S5. — The expression patterns of HSFs and DREBs under stress conditions revealed by RNA-seq data. The expression trends of HSFs and DREBs determined by RNA-seq and qRT-PCR are consistent, indicating the high confidence of RNA-seq data. [file 12870_2015_511_MOESM2_ESM.tiff]

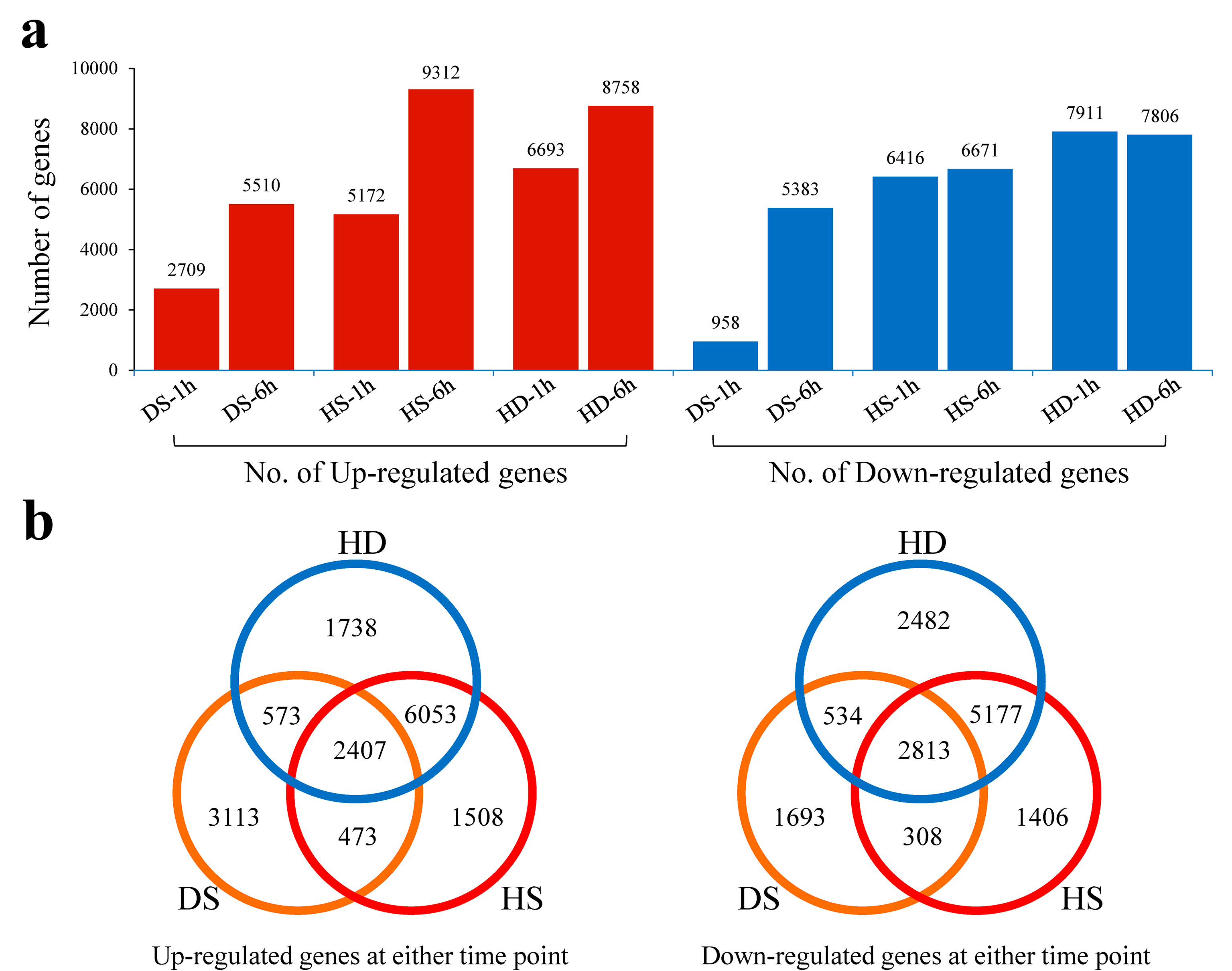

Supplement: Additional file 13: Fig. S6. — Flowchart of homeologous gene expression analysis. (a) Identification of triplets based on wheat reference genes released by IWGSC. In total, 4,565 triplets were identified based on our criteria, and 2,804 were differentially expressed when subjected to DS, HS or HD. (b) Expression analysis of A-, B- and D-homeologs. Reads mapped to a triplet can be classified into 10 groups based on SNP information and the formulas used to calculate each homeolog’s expression are shown. [file 12870_2015_511_MOESM4_ESM.tiff]

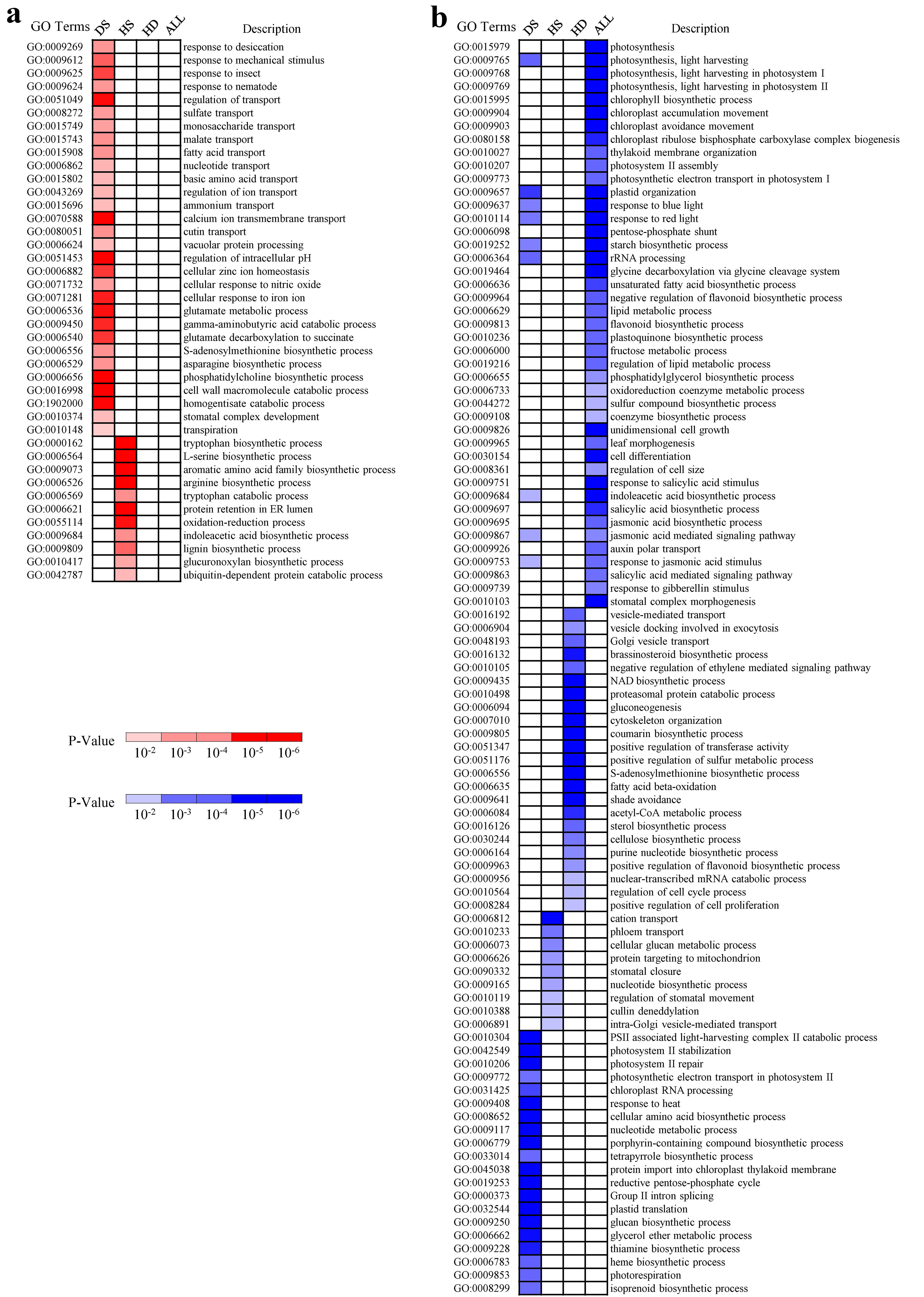

Supplement: Additional file 14: Table S8. — Details of 2,804 differentially expressed triplets. [file 12870_2015_511_MOESM5_ESM.tiff]

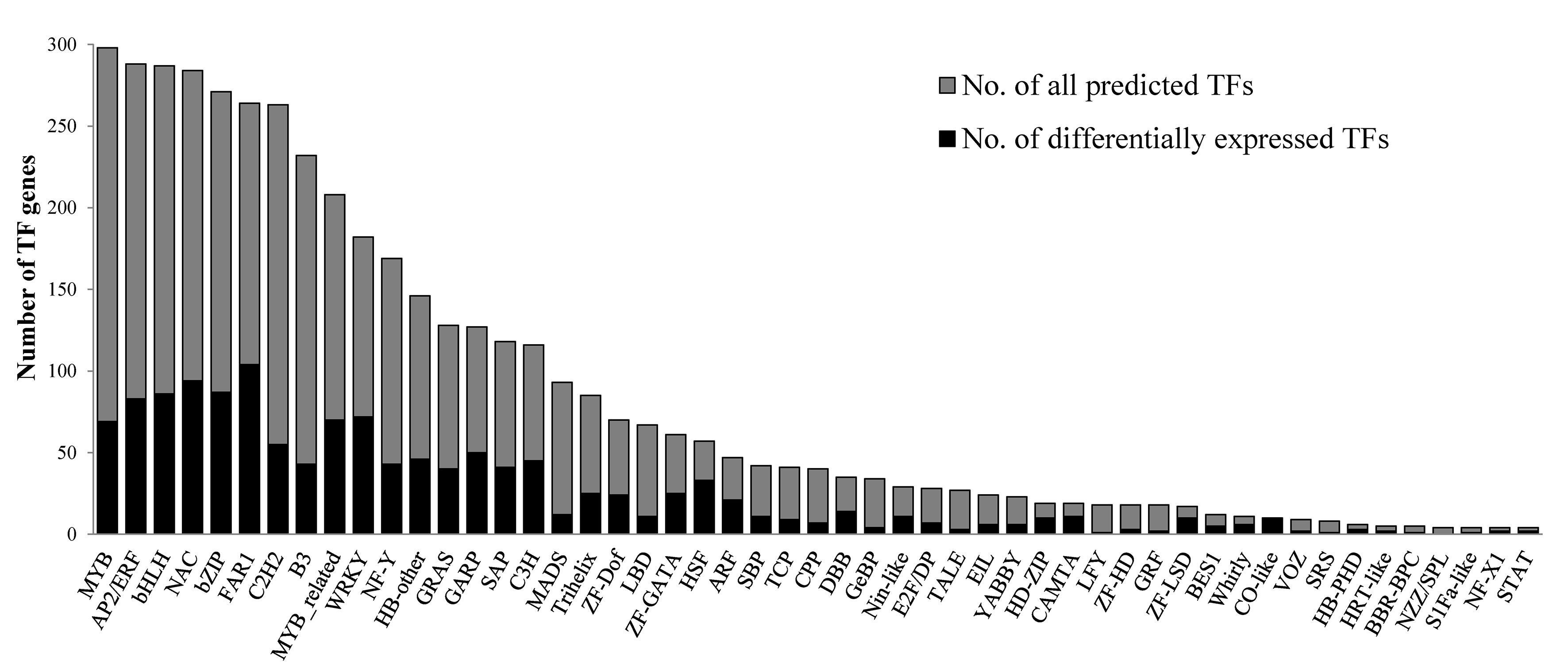

Supplement: Additional file 15: Fig. S7. — Clustering analysis of stress-related homeologs with differential responses. (a) Triplets showing differential responses between homeologs can be clustered into 12 clusters based on homeologs responsive patterns. The red and blue line of each chart represents the average responsive trend of up- and down-regulated homeologs, respectively. (b) Statistics of the numbers of triplets within the 12 clusters. [file 12870_2015_511_MOESM6_ESM.tiff]
